# AI/ML combined with next-generation sequencing of VHH immune repertoires enables the rapid identification of *de novo* humanized and sequence-optimized single domain antibodies: a prospective case study

**DOI:** 10.3389/fmolb.2023.1249247

**Published:** 2023-09-28

**Authors:** Paul Arras, Han Byul Yoo, Lukas Pekar, Thomas Clarke, Lukas Friedrich, Christian Schröter, Jennifer Schanz, Jason Tonillo, Vanessa Siegmund, Achim Doerner, Simon Krah, Enrico Guarnera, Stefan Zielonka, Andreas Evers

**Affiliations:** ^1^ Antibody Discovery and Protein Engineering, Merck Healthcare KGaA, Darmstadt, Germany; ^2^ Institute for Organic Chemistry and Biochemistry, Technical University of Darmstadt, Darmstadt, Germany; ^3^ Bioinformatics, EMD Serono, Billerica, MA, United States; ^4^ Computational Chemistry and Biologics, Merck Healthcare KGaA, Darmstadt, Germany; ^5^ ADCs & Targeted NBE Therapeutics, Merck KGaA, Darmstadt, Germany; ^6^ Early Protein Supply and Characterization, Merck Healthcare KGaA, Darmstadt, Germany

**Keywords:** artificial intelligence and machine learning (ML), deep learning, *in silico* developability, long short-term memory (LSTM), next-generation sequencing (NGS), single domain antibodies (VHH), yeast surface display (YSD), protein engineering

## Abstract

**Introduction:** In this study, we demonstrate the feasibility of yeast surface display (YSD) and nextgeneration sequencing (NGS) in combination with artificial intelligence and machine learning methods (AI/ML) for the identification of de novo humanized single domain antibodies (sdAbs) with favorable early developability profiles.

**Methods:** The display library was derived from a novel approach, in which VHH-based CDR3 regions obtained from a llama (Lama glama), immunized against NKp46, were grafted onto a humanized VHH backbone library that was diversified in CDR1 and CDR2. Following NGS analysis of sequence pools from two rounds of fluorescence-activated cell sorting we focused on four sequence clusters based on NGS frequency and enrichment analysis as well as in silico developability assessment. For each cluster, long short-term memory (LSTM) based deep generative models were trained and used for the in silico sampling of new sequences. Sequences were subjected to sequence- and structure-based in silico developability assessment to select a set of less than 10 sequences per cluster for production.

**Results:** As demonstrated by binding kinetics and early developability assessment, this procedure represents a general strategy for the rapid and efficient design of potent and automatically humanized sdAb hits from screening selections with favorable early developability profiles.

## Introduction

VHHs (variable domain of the heavy chain of a heavy chain-only antibodies), commercially known as nanobodies, are single-domain antibody (sdAb) fragments derived from camelid heavy chain-only antibodies (HcAbs). VHHs exhibit small size, high stability, and exceptional binding specificity, making them valuable tools for therapeutics, diagnostics, and research applications (Krah et al., 2016; Könning et al., 2017; Wang et al., 2022; Jin et al., 2023). Owing to their simple molecular architecture, they offer a plethora of engineering options with respect to the generation of bi- and multispecific antibody designs involving different paratope valences and spatial orientations of individual domains within a given molecule (Bannas et al., 2017; Chanier and Chames, 2019; Pekar et al., 2020; Yanakieva et al., 2022; Lipinski et al., 2023a; Lipinski et al., 2023b). However, VHH domains usually have to be humanized and further sequence-optimized to be suitable for therapeutic applications.

A classical cascade for antibody and VHH discovery typically involves (camelid) immunization and antibody library construction after immunization followed by antibody selections or panning. Subsequently, Sanger sequencing of high prevalent clones can be applied (typically in the range of a couple of hundred clones) that are then profiled for the desired on-target effect, and functional or phenotypic assays. The best hits are then nominated for sequence optimization, usually including humanization (Vincke et al., 2009; Sulea et al., 2022), replacement of chemically labile and post-translational modification (PTM) motifs and ideally considering further developability-related aspects (Lauer et al., 2012; Sormanni et al., 2015; Raybould et al., 2019; Ahmed et al., 2021; Khetan et al., 2022; Negron et al., 2022; Evers et al., 2023a; Fernández-Quintero et al., 2023; Jain et al., 2023; Mieczkowski et al., 2023; Svilenov et al., 2023). Sometimes, the complexity of these different optimization parameters might require multiple design cycles and in some cases it might not be even possible to optimize such hits towards a favorable overall profile (Rabia et al., 2018). This process of iterative sequence optimization is generally on the critical path in early biologics drug discovery projects. Therefore, it is highly desirable to find new approaches that accelerate the discovery and design of humanized sequences with a favorable early developability profile, both in terms of project timelines and to reduce attrition in the downstream process.

In contrast to the traditional approach of Sanger sequencing, next-generation sequencing (NGS) of screening pools obtained from selection campaigns enables a rapid and cost-effective analysis of the vast sequence spaces of binders (Larman et al., 2012; Mathonet and Ullman, 2013; Hu et al., 2015; Barreto et al., 2019). Integration of Sequence-Activity-Relationship (SAR), frequency and enrichment analyses with *in silico* developability assessment on NGS data can furthermore provide a rational approach to identify potent sequences with improved developability profiles. Moreover, recent studies have shown the versatility of artificial intelligence/machine learning (AI/ML) techniques on antibody NGS data to design new sequences with potentially further improved potency or developability (Liu et al., 2020; Mason et al., 2021; Saka et al., 2021; Makowski et al., 2022; Hie et al., 2023; Parkinson et al., 2023). In these studies, regions of specific antibody candidates were diversified in combinatorial mutagenesis display libraries, followed by the generation of ML models from NGS data. Saka et al. (2021), for example, employed long short-term memory (LSTM) based on NGS derived sequences from different panning rounds of a library diversified in CDR-H1, -H2 and -H3 and FR1 of a kynurenine binding antibody. The affinities of newly designed sequences were over 1800-fold higher than for the parental clone. LSTM is a widely used deep learning architecture in natural language processing that is also particularly effective in predicting new protein sequences, as it is capable of modeling long-term dependencies and capturing the complex relationships between amino acids that determine structure and function. Such LSTMs have not only been successfully applied for the design of new antibodies (Saka et al., 2021), but also for peptides (Müller et al., 2018) and small molecules (Gupta et al., 2018; Merk et al., 2018; Segler et al., 2018; Z et al., 2022). While the above-mentioned studies used combinatorial synthetic display libraries in combination with NGS and AI/ML to optimize existing lead antibodies, this concept might also be employed to discover new and potent antibody sequences with favorable developability profiles from diverse antibody repertoires obtained from animal immunization.

As part of our integrated VHH hit discovery strategy, we have recently implemented a semi-immune/semi-synthetic library approach for the high-throughput *de novo* identification of humanized VHHs following camelid immunization (Arras et al., 2023). For this, VHH-derived CDR3 regions obtained from a llama, immunized against recombinant human (rh) Natural Cytotoxicity Receptor NKp46 (Barrow et al., 2019), were grafted onto a humanized VHH backbone library comprising sequence-diversified CDR1 and CDR2 regions that were tailored towards favorable *in silico* developability properties, by considering human-likeness and excluding potential sequence liabilities and predicted immunogenic motifs. NKp46 is an activating receptor on Natural Killer cells (NK cells) and was successfully harnessed for the generation of potent NK cell engagers (Gauthier et al., 2019; Gauthier et al., 2023; Lipinski et al., 2023). Target-specific humanized VHHs were readily obtained in our previous study by YSD (Arras et al., 2023). By exploiting this approach, high affinity VHHs with optimized developability profiles can principally be generated against any antigen of interest upon camelid immunization. The process of CDR3 engraftment onto our generic humanized and sequence-optimized VHH scaffold library is characterized by its low complexity and duration similar to the generation of wild-type VHH display libraries following immunization (Roth et al., 2020); thereby this procedure significantly accelerates VHH hit discovery by reducing or even eliminating the need for subsequent sequence optimization. Due to the setup of our library approach, all resulting VHHs have a fixed humanized framework sequence, e.g., any differences in antigen binding and developability properties are driven by sequence variations in the CDR regions. Providing NGS data from different rounds of YSD (Valldorf et al., 2022) based FACS screens from this library therefore represent ideal inputs to train AI/ML models for the design of new sequences with even further improved potency and developability.

Goal of the present study was to investigate the feasibility of our integrated approach of combining i) camelid immunization, ii) humanized VHH library generation, iii) YSD, iv) FACS screening, v) NGS analysis, vi) AI/ML based sequence sampling and vii) *in silico* developability assessment to identify potent and readily sequence optimized VHH hits in a single procedure. The display library was derived from our humanized VHH library that was directed against (rh) NKp46 (Arras et al., 2023). Based on NGS analysis, we selected four diverse CDR3 sequence clusters in the present study that showed high frequency or enrichment over two rounds of FACS screening. These repertoires were used to train LSTM deep generative models for the automated design of new sequences that were subsequently filtered based on *in silico* developability criteria using our recently described *Sequence Assessment Using Multiple Optimization criteria* (SUMO) approach (Evers et al., 2023a). We finally selected a set of only up to ten sequences per cluster for synthesis and experimental profiling. As demonstrated in binding measurements and early developability assays, the proposed methodology has the capability to generate diverse and potent VHH hits directly from screening collections upon camelid immunization that do ideally not require further humanization and sequence optimization. Furthermore, it provides sequence activity (SAR) and sequence-property (SPR) relationships for each of the investigated sequence clusters. Taken together, as exemplified and demonstrated on a typical early drug discovery project, this workflow has the potential to significantly accelerate hit discovery and optimization and reduce the risk for developability-related attrition.

## Results

### Previous work: humanized VHH library construction after camelid immunization, yeast surface display and cell sorting

**FIGURE 1:**
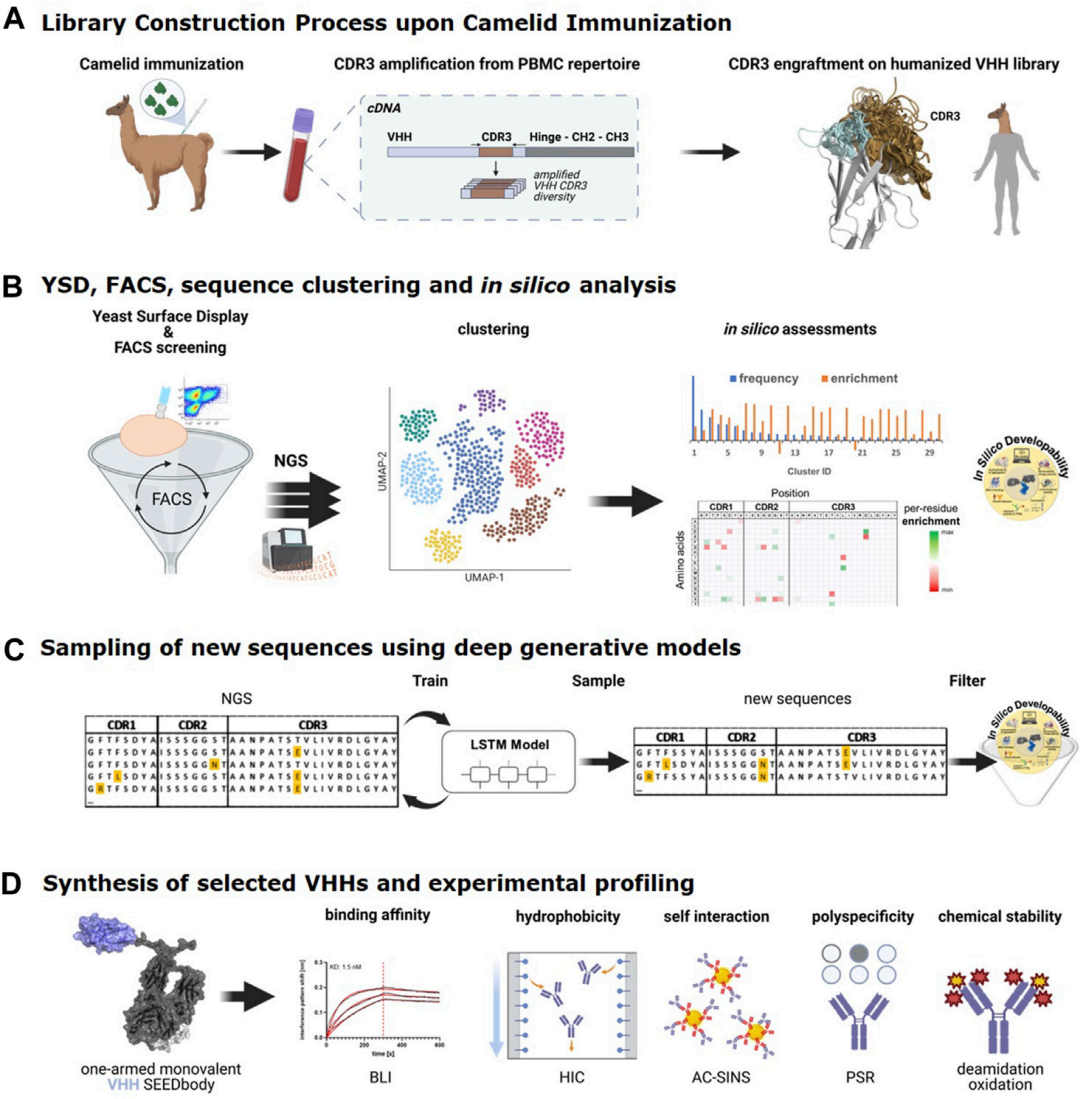
The end-to-end process consists of the following steps: **(A)**. Library construction process. VHH-derived CDR3 regions obtained from a llama, immunized against (rh) NKp46 are grafted onto a generic humanized and sequence-optimized VHH backbone library. **(B)**. Process of binder identification from Yeast Display Library based on multiple rounds of FACS and next-generation sequencing (NGS) analysis of sequence pools before and after FACS, followed by sequence clustering, per-cluster frequency and enrichment analyses in combination with *in silico* developability predictions to identify most interesting sequence clusters. **(C)**. Per-cluster LSTM deep generative model generation and sampling of new sequences that are subjected to *in silico* developability assessment to identify sequences for synthesis and experimental profiling. **(D)**. Selected VHH sequences are produced as one-armed monovalent SEEDbodies and experimentally characterized for binding against NKp46 and in early developability assays. (Figures partially created with BioRender.com).

As outlined in detail in our previous study (Arras et al., 2023) and schematically illustrated in Figure 1A, we have recently developed a semi-immune/semi-synthetic strategy that relies on grafting the PBMC-amplified CDR3 VHH repertoire of llamas following immunization onto two internally optimized humanized backbone libraries with a framework germline sequence derived from human IGHV3-23*1 (Arras et al., 2023). Both libraries were diversified in CDR1 and CDR2 towards favorable *in silico* developability properties, i) considering amino acid distributions observed in naïve and immunized llamas, eliminating residue combinations ii) that would result in potential N-glycosylation sites (Asn-X-Ser/Thr) or highly susceptible chemical liability motifs (Asn-Gly, Asp-Gly, Met, unpaired Cys) and iii) strong predicted MHC-II binding peptide motifs, while taking into account iv) diversity with respect to charge, size and hydrophobicity and v) occurrence in the equivalent positions in NGS data of human antibody repertoires. To identify novel binders against (rh) NKp46, we had opted for PBMC-derived total RNA of a (rh) NKp46 immunized llama for the generation of both CDR3-engrafted humanized libraries for YSD. As demonstrated in a head-to-head comparison, sequences from the CDR3-engrafted humanized library that were selected after two rounds of FACS showed similar activity against NKp46 compared to CDR3-analogues from immunized WT llama sequences with improved early developability profiles (Arras et al., 2023). In that study, 96 clones were selected after FACS by random picking and Sanger sequencing from each library. For the present study, we re-analyzed the sequence pools of the CDR3-engrafted humanized library from the different selection rounds by NGS (Figure 1B).

### Identification of sequence clusters based on NGS analysis and *in silico* developability assessment

The application of NGS in combination with AI/ML approaches can represent a quick and cost-effective way to identify potent and developable binders that might not be picked with the traditional approach of random clone selection and Sanger sequencing. To exhaustively assess sequence diversity from our previous display campaign, NGS data for screening pools obtained from the different FACS rounds of the CDR3-engrafted humanized library were generated using the MiSeq system (Figure 1B). Table 1 summarizes the absolute number of NGS reads that were obtained after the different rounds of FACS for all sequences and for those CDR3 sequence clusters that were used for LSTM deep generative model generation as outlined below.

**TABLE 1:** Summary of NGS data. VHH genes of screening samples were analyzed using MiSeq. Sequences were clustered based on 50% CDR3 sequence identity. Number of NGS reads are shown for all sequences and for those clusters that were selected for sampling of new sequences, antibody production and experimental profiling based on enrichment analysis and *in silico* developability assessment. Sequences obtained from FACS round 2 were used for LSTM deep generative model generation.

clusterID	NGS reads			Enrichment factor round 2 vs. round 0
	FACS round 0	FACS round 1	FACS round 2	
1	0	942	2,630	3,095
2	1	2,790	2,991	1760
3	36	4,964	4,147	132
4	888	8,573	11,954	16
ALL	887,881	1,138,880	754,669	

Sequences were annotated with Geneious Biologics (Antibody Discovery Software, 2023) using IMGT numbering and clustered based on 50% CDR3 sequence identity. We assumed that this cutoff assures that i) within each cluster most VHHs bind in a similar manner to the same epitope, and ii) at the same time provides sufficient sequence diversity within each cluster for ML model generation, SAR analysis and automated multi-parameter optimization towards improved potency and developability. All sequence clusters were ranked by either i) their absolute frequency (total number of reads), i.e., the number of clones observed after the second round of FACS or ii) their enrichments (as described in Materials and Methods) observed over FACS round 2 vs. round 0 (Figure 1B; Table 1). The ranking of clusters and sequences based on their absolute frequency should principally result in similar selections compared to the random selection and Sanger sequencing approach that is usually applied in the traditional screening cascade. Conversely, selection based on enrichment is potentially able to identify rare clones with superior affinity and specificity (Rouet et al., 2018; Barreto et al., 2019). In a first feasibility study, we selected the most occurring CDR1-3 amino acid sequence from the i) five most frequent and ii) five most enriched CDR3 clusters for production and binding affinity determination against NKp46. Since two CDR3 clusters occurred in both sets, a total of eight sequences were produced and tested (Table 2). Remarkably, seven sequences showed binding affinity in the 1-digit nanomolar range. Only the representative of the most frequent cluster exhibits a slightly lower binding affinity (KD = 19.8 nM). These results are in agreement with previous literature reports that enrichment-based selection based on NGS data can provide additional potent sequences (Rouet et al., 2018; Barreto et al., 2019).

**TABLE 2:** Most occurring CDR1-3 sequences and binding affinity data against NKp46 of the five i) most enriched and five ii) most frequent clusters that were obtained from NGS of sequence pools obtained from YSD-FACS. Of note, two CDR3 clusters occurred in both sets. Hence a total of eight sequences were produced and tested. To visualize sequence diversity, amino acid differences to the most frequent residue in each position are shown in orange boxes.

cluster ranking			CDR1-3 sequence																																			
most enriched	most frequent	KD [nM]	CDR1								CDR2								CDR3																			
1		1.1	G	G	T	F	G	N	Y	A	I	S	R	G	G	G	S	T	A	A	A	G	G	M	G	S	T	T	V	V	V	S	T	I	P	Y	K	Y
2		8.6	G	F	T	F	S	S	Y	A	I	S	S	S	G	S	N	T	A	A	V	F	T	P	T	D	T	V	V	F	T	N	K	E	P	Y	N	Y
3		31.7	G	F	T	F	G	N	Y	A	I	S	S	S	G	D	S	T	A	A	V	E	A	D	S	S	E	V	V	F	L	S	P	H	I	Y	Q	Y
4	4	2.3	G	R	T	F	G	N	Y	A	I	S	R	S	G	G	S	T	A	A	N	P	A	T	S	T	V	-	L	I	V	R	D	L	G	Y	A	Y
5	5	1.7	G	F	T	F	S	S	Y	A	I	S	G	S	G	D	S	T	A	A	D	Q	Q	P	P	S	V	-	A	V	V	A	A	R	G	Y	R	Y
	3	3.1	G	F	T	F	S	D	Y	A	I	S	S	S	G	G	N	T	A	T	S	L	T	Y	D	Q	T	T	V	Y	V	S	P	L	A	Y	V	D
	2	4.0	G	R	T	L	S	S	Y	V	I	S	S	S	G	D	R	T	A	A	A	L	A	P	S	G	T	L	V	V	V	S	P	L	G	Y	T	Y
	1	19.8	G	R	T	F	S	S	Y	A	I	S	S	S	G	G	S	T	A	A	I	R	T	P	A	E	S	Q	V	I	V	T	L	D	W	Y	R	Y

**FIGURE 2:**
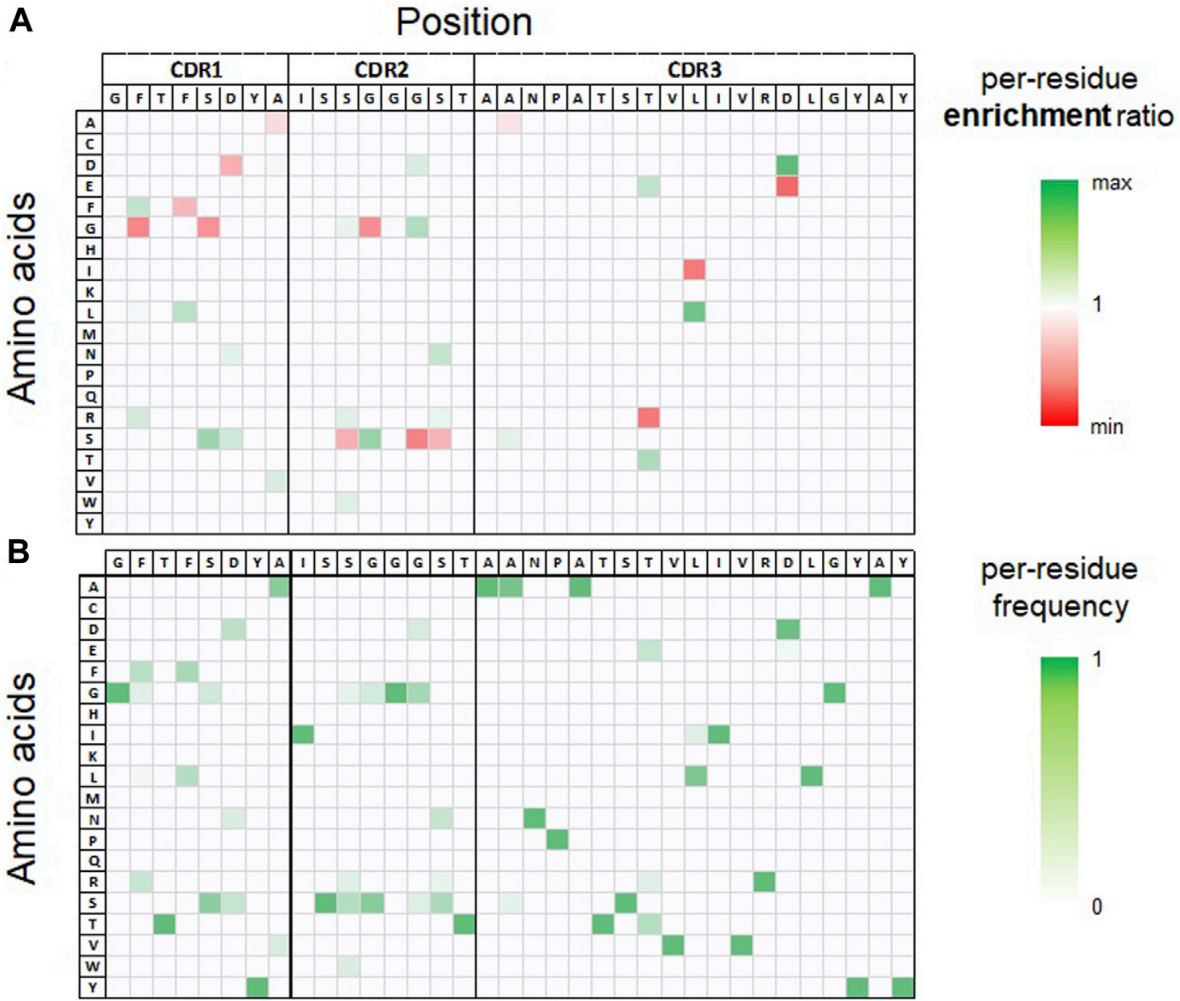
Per-residue enrichment and frequency analysis, both illustrated as heat-map for CDR3 sequence cluster 3. The table headers show the CDR1-3 sequence of the most frequent clone observed in the NGS data set after the second round of FACS selection within this cluster. **(A)**. Per-residue enrichment ratio over YSD-FACS rounds 1–2. Residues with a high enrichment (colored green) are observed with a higher relative frequency after FACS round 2 compared to round 1. **(B)**. Per-residue frequency distribution observed after FACS round 2.

As mentioned above, due to our library design strategy, all sequences are identical in their framework regions that were derived from a humanized germline sequence. In the next step, we analyzed the sequence and computed property space within each CDR3 sequence cluster. To visualize diversity (based on sequence identity) after each round of FACS enrichment, the respective sequences pools were projected into a two-dimensional space using UMAP (Becht et al., 2018) (Supplementary Figure S1). In addition, i) the per-residue frequency distributions of clones obtained after the second round of FACS and ii) the per-residue enrichment ratio through FACS enrichment rounds 1–2 were computed and analyzed, as shown in Figure 2 and Supplementary Figures S2–S4. Finally, for each cluster the 100 most frequent unique sequences obtained from FACS round 2 were subjected to *in silico* developability assessment using our previously described SUMO approach (Evers et al., 2023a). This method automatically generates structural VHH models from provided sequences, evaluates their human-likeness, and identifies potential surface-exposed chemical liabilities and post-translational modification motifs. Additionally, a small set of computed physico-chemical descriptors is reported, including the isoelectric point (pI), AggScore (Sankar et al., 2018) as predictor for hydrophobicity and aggregation tendency, and the positive patch energy of the CDRs. Analysis of sequence and predicted property data was used to assess the sequence spaces within each cluster regarding their potential to provide i) potent sequences, ii) broad sequence diversity and SAR information and iii) favorable *in silico* developability properties. We were particularly interested in selecting clusters with considerable sequence diversity to investigate how LSTM sampling could provide new sequence combinations to increase diversity and ideally improve affinity and/or developability properties. Based on these analyses, we picked four sequence clusters (termed cluster IDs 1–4 in the following) for LSTM based deep generative model generation and sampling of new sequences. The original data files used for sequence and *in silico* property analysis are provided in Supplementary Tables S1–S4 and illustrated for CDR cluster 3 in Supplementary Figures S5, S6.

### LSTM model structure, training, sequence generation and scoring

As illustrated in Figure 1C, the LSTM model training and design was conducted using a recurrent network structure that has previously been successfully applied for the design of peptides [details in ref (Müller et al., 2018)]. LSTM models capture patterns in sequential data and generate new data instances from the learned distributions. Like their utility in peptide applications, the amino acid sequences of VHHs serve as appropriate inputs for these machine learning models. Since all sequences of the current study have identical framework regions, only the CDR1-3 sequences were concatenated and used for the training of LSTM models. For each of the four selected CDR3 sequence clusters, these CDR1-3 sequences (including all redundant sequences) from the second FACS round (Table 1) as determined by NGS were used for training. The best models were selected by evaluating the calculated validation losses on the left-out training datasets using a five-fold cross-validation approach (Supplementary Figure S7). Based on the learning distribution of the trained LSTM models, new sequences were sampled. We sampled 10,000 new sequences per cluster. These new sequences were combined with the original training sequences and ranked by their calculated *negative logarithm of likelihood* (NLL), a score that reflects the observed frequency of individual amino acids along the sequences of the training data sets (see Methods, Supplementary Figure S5 and Supplementary Tables S1–S4). The NLL score is not a predictor for binding affinity *per se*. However, since it reflects the sequence bias of amino acid distributions in the training data set sorted for favorable binding by FACS, it has been shown to represent a pragmatic score for selecting new sequences with an increased likelihood for high binding affinity (Saka et al., 2021).

### *In silico* developability assessment to identify sequences for production and experimental profiling

Within each cluster, the top-ranked 100 non-redundant sequences obtained from LSTM sampling and NGS analysis were subjected to *in silico* developability assessment (see Supplementary Tables S1–S4) using our SUMO approach (Evers et al., 2023a). With the available sequences and their *in silico* profiles, the primary goal was to select a set of ≤10 sequences (for each cluster) for synthesis from which at least one sequence (per cluster) should be suited for further project progression after experimental profiling without the need for further iterative sequence optimization. For the nomination of these sequences, the following criteria were taken into account.

1. NLL scores: To assess the NLL’s effectiveness in estimating binding affinities, we chose binders within each cluster with highly favorable scores, nominating at least three sequences from the top 100 scoring sequences. Additionally, we intentionally selected further sequences beyond the top 100 to cover a broad range of NLL scores, facilitating subsequent correlation analyses with experimental binding affinities.
2. *In silico* developability criteria: To minimize the risk of aggregation and non-specific binding, we selected sequences with computed aggregation propensity and positive charged CDR patch scores below defined cutoff scores. These cutoffs were set to the computed average scores plus one standard deviations over a data set of 79 marketed antibodies (see Table 4 legend). Additionally, as general de-risking approach, we intentionally picked sequence variants covering a certain pI range (Supplementary Table S5). The pI of an antibody/VHH can significantly impact various developability properties, such as solubility, aggregation during purification, virus inactivation (Jin et al., 2019), colloidal stability, viscosity in formulation (Kingsbury et al., 2020; Gupta et al., 2022), or non-specific binding or clearance (Ahmed et al., 2021; G et al., 2021). Small sequence modifications have been shown to improve colloidal stability and viscosity behavior (Kumar et al., 2018; Evers et al., 2019). Considering that the optimal pI for an antibody drug product may vary depending on environmental factors, such as a solution or formulation pH, often not yet defined in the early project phase, selecting additional pI variants of a lead sequence provides potential backups for efficient project progression and de-risking.
3. Sequence diversity within each CDR3 cluster for SAR generation and chemical liability site elimination: Our humanized VHH library design strategy (Arras et al., 2023) omits N-glycosylation sites (Asn-X-Ser/Thr) and highly susceptible chemical liability sites (Asn-Gly, Asp-Gly, Met, Cys) in CDR1 or CDR2 (Table 3). However, such liabilities may still occur in CDR3, which is directly grafted from NKp46-immunized llama VHHs. Additionally, other theoretical chemical liability motifs (e.g., Asn-Ser, Asn-Asn, Asn-Thr; Asp-Ser, Asp-Asp, Asp-Thr, *etc.*) may be present in CDR1 or CDR2. These had not been excluded from library design, since degradation of these motifs occurs significantly less frequently based on internal and literature data (Lu et al., 2019) and are therefore assessed case-by-case, either by post-filtering based on more rigorous *in silico* liability assessments or by experimental profiling as exemplified below. As shown in Table 3, several selected sequences possess such “less severe” liability motifs. As part of our de-risking strategy, we intentionally selected sequence variants within each cluster where residues theoretically prone to chemical degradation (e.g., Asn, Asp, Met) are replaced by chemically non-reactive residues (e.g., sequences 15–17, where a Met residue in CDR3 is replaced by Ile).
4. Finally, we ensured that for all four clusters, sequences were selected from both the NGS output and LSTM sampled sequences to assess, through experimental profiling, the extend to which LSTM sampling provided additional or improved “chemical matter”.

**TABLE 3:** CDR1-3 sequences of VHHs obtained from NGS analysis and AI/ML (LSTM) predictions. Sequences are grouped by their CDR3 cluster ID (50% SEQ-ID cutoff) with the most potent sequence at the top of each group. To visualize sequence and property relationships, amino acid differences to the most potent sequence within each group are shown in orange boxes. Residues that might theoretically be prone to chemical degradation are colored red (Asn deamidation, Asp isomerization, Met oxidation). In addition, the predicted NLL score and experimentally measured binding affinities (KD) as well as the k_on_ and k_off_ values are provided. NB: no binding.

ID	CDR3 cluster	source	KD [nM]	kon [1/Ms]	koff [1/s]	NLL	CDR1								CDR2								CDR3																			
1	1	AI/ML	5.3	3.2E+05	1.7E-03	4.9	**G**	**R**	**T**	**F**	**S**	N	**Y**	**A**	**I**	**S**	**R**	**G**	**G**	**D**	N	**T**	**A**	**A**	**V**	**F**	**T**	**P**	**T**	D	**T**	**V**	**V**	**F**	**I**	N	**K**	**E**	**P**	**Y**	N	**Y**
2	1	NGS	7.4	3.4E+05	2.5E-03	4.8	**G**	**F**	**T**	**F**	**S**	**S**	**Y**	**A**	**I**	**S**	**S**	**S**	**G**	**S**	N	**T**	**A**	**A**	**V**	**F**	**T**	**P**	**T**	D	**T**	**V**	**V**	**F**	**T**	N	**K**	**G**	**P**	**Y**	N	**Y**
3	1	AI/ML	8.1	6.9E+04	5.6E-04	6.2	**G**	**R**	**T**	**F**	**S**	**S**	**Y**	**A**	**I**	**S**	**R**	**G**	**G**	**D**	N	**T**	**A**	**A**	**V**	**F**	**T**	**P**	**T**	D	**T**	**V**	**V**	**F**	**I**	N	**K**	**E**	**S**	**Y**	N	**Y**
4	1	NGS	8.6	1.3E+05	1.1E-03	2.9	**G**	**F**	**T**	**F**	**S**	**S**	**Y**	**A**	**I**	**S**	**S**	**S**	**G**	**S**	N	**T**	**A**	**A**	**V**	**F**	**T**	**P**	**T**	D	**T**	**V**	**V**	**F**	**T**	N	**K**	**E**	**P**	**Y**	N	**Y**
5	1	NGS	9.4	1.9E+05	1.8E-03	2.5	**G**	**F**	**T**	**F**	**S**	**S**	**Y**	**A**	**I**	**S**	**S**	**G**	**G**	**D**	**S**	**T**	**A**	**A**	**V**	**F**	**T**	**P**	**T**	D	**T**	**V**	**V**	**F**	**T**	N	**K**	**E**	**P**	**Y**	N	**Y**
6	1	AI/ML	11.3	1.8E+05	2.1E-03	2.5	**G**	**F**	**T**	**F**	**S**	**S**	**Y**	**A**	**I**	**S**	**S**	**S**	**G**	**G**	**S**	**T**	**A**	**A**	**V**	**F**	**T**	**P**	**T**	D	**T**	**V**	**V**	**F**	**T**	N	**K**	**E**	**P**	**Y**	N	**Y**
7	1	NGS	11.7	2.3E+05	2.7E-03	2.8	**G**	**R**	**T**	**F**	**S**	**S**	**Y**	**A**	**I**	**S**	**S**	**S**	**G**	**G**	**S**	**T**	**A**	**A**	**V**	**F**	**T**	**P**	**T**	D	**T**	**V**	**V**	**F**	**T**	N	**K**	**E**	**P**	**Y**	N	**Y**
8	1	AI/ML	13.9	1.9E+05	2.7E-03	2.7	**G**	**F**	**T**	**L**	**S**	**S**	**Y**	**A**	**I**	**S**	**S**	**G**	**G**	**G**	**S**	**T**	**A**	**A**	**V**	**F**	**T**	**P**	**T**	D	**T**	**V**	**V**	**F**	**T**	N	**K**	**E**	**P**	**Y**	N	**Y**
9	1	AI/ML	21.9	5.4E+04	1.2E-03	20.8	**G**	**G**	**T**	**F**	**S**	**I**	**Y**	**A**	**I**	**S**	**R**	**G**	**G**	**S**	N	**T**	**A**	**A**	**V**	**F**	**T**	**P**	**T**	D	**T**	**V**	**V**	**F**	**I**	N	**K**	**E**	**R**	**Y**	N	**Y**
10	2	AI/ML	<0.1	1.9E+05	<1.0E-07	2.4	**G**	**G**	**T**	**F**	**G**	**S**	**Y**	**A**	**I**	**S**	**R**	**S**	**G**	**G**	**S**	**T**	**A**	**A**	**A**	**G**	**G**	M	**G**	**S**	**T**	**T**	**V**	**V**	**V**	**S**	**T**	**I**	**P**	**Y**	**K**	**Y**
11	2	NGS	0.8	2.3E+05	2.0E-04	2.3	**G**	**G**	**T**	**F**	**S**	**S**	**Y**	**A**	**I**	**S**	**S**	**S**	**G**	**G**	**S**	**T**	**A**	**A**	**A**	**G**	**G**	M	**G**	**S**	**T**	**T**	**V**	**V**	**V**	**S**	**T**	**I**	**P**	**Y**	**K**	**Y**
12	2	NGS	1.1	2.5E+05	2.8E-04	2.8	**G**	**G**	**T**	**F**	**G**	N	**Y**	**A**	**I**	**S**	**R**	**G**	**G**	**G**	**S**	**T**	**A**	**A**	**A**	**G**	**G**	M	**G**	**S**	**T**	**T**	**V**	**V**	**V**	**S**	**T**	**I**	**P**	**Y**	**K**	**Y**
13	2	NGS	1.3	2.0E+05	2.7E-04	2.3	**G**	**G**	**T**	**F**	**S**	**S**	**Y**	**A**	**I**	**S**	**R**	**S**	**G**	**G**	**S**	**T**	**A**	**A**	**A**	**G**	**G**	M	**G**	**S**	**T**	**T**	**V**	**V**	**V**	**S**	**T**	**I**	**P**	**Y**	**K**	**Y**
14	2	AI/ML	1.5	3.6E+05	5.3E-04	2.6	**G**	**G**	**T**	**F**	**S**	N	**Y**	**A**	**I**	**S**	**S**	**S**	**G**	**G**	**S**	**T**	**A**	**A**	**A**	**G**	**G**	M	**G**	**S**	**T**	**T**	**V**	**V**	**V**	**S**	**T**	**I**	**P**	**Y**	**K**	**Y**
15	2	NGS	2.3	1.1E+05	2.6E-04	7.1	**G**	**R**	**T**	**F**	**G**	**S**	**Y**	**A**	**I**	**S**	**S**	**S**	**G**	D	**S**	**T**	**A**	**A**	**A**	**G**	**G**	**I**	**G**	**S**	**S**	**T**	**V**	**V**	**V**	**S**	P	**I**	**P**	**Y**	**A**	**Y**
16	2	NGS	4.0	1.6E+05	6.3E-04	8.4	**G**	**R**	**T**	**L**	**S**	**S**	**Y**	**V**	**I**	**S**	**S**	**S**	**G**	D	**R**	**T**	**A**	**A**	**A**	**L**	A	P	**S**	**G**	**T**	**L**	**V**	**V**	**V**	**S**	P	**L**	**G**	**Y**	**T**	**Y**
17	2	AI/ML	4.4	1.0E+05	4.5E-04	2.8	**G**	**G**	**T**	**F**	**G**	N	**Y**	**A**	**I**	**S**	**R**	**G**	**G**	**G**	**S**	**T**	**A**	**A**	**A**	**G**	**G**	**I**	**G**	**S**	**T**	**T**	**V**	**V**	**V**	**S**	**T**	**I**	**P**	**Y**	**K**	**Y**
18	2	AI/ML	NB	NB	NB	17.8	**G**	**G**	**T**	**F**	**G**	N	**Y**	**A**	**I**	**S**	**R**	**G**	**G**	**G**	**S**	**T**	**A**	**A**	**A**	**G**	**G**	M	**G**	**S**	**T**	**T**	**V**	**V**	**V**	**S**	**T**	**I**	**P**	P	**K**	**Y**
19	2	AI/ML	NB	NB	NB	17.8	**G**	**G**	**T**	**F**	**G**	N	**Y**	**A**	**I**	**S**	**R**	**G**	**G**	**G**	**S**	**T**	**A**	**A**	**A**	**G**	**G**	M	**G**	**S**	**T**	**T**	E	**V**	**V**	**S**	**T**	**I**	**P**	**Y**	**K**	**Y**
20	3	AI/ML	2.1	1.3E+05	2.8E-04	3.6	**G**	**G**	**T**	**F**	**S**	D	**A**	**A**	**I**	**S**	**R**	**S**	**G**	D	**S**	**T**	**A**	**A**	N	**P**	**A**	**T**	**S**	**E**	**V**	**L**	**I**	**V**	**R**	D	**L**	**G**	**Y**	**A**	**Y**	
21	3	NGS	2.3	1.4E+05	3.2E-04	3.0	**G**	**R**	**T**	**F**	**G**	N	**Y**	**A**	**I**	**S**	**R**	**S**	**G**	**G**	**S**	**T**	**A**	**A**	N	**P**	**A**	**T**	**S**	**T**	**V**	**L**	**I**	**V**	**R**	D	**L**	**G**	**Y**	**A**	**Y**	
22	3	AI/ML	4.9	1.2E+05	5.8E-04	3.2	**G**	**R**	**T**	**F**	**S**	**S**	**Y**	**A**	**I**	**S**	**S**	**S**	**G**	**G**	N	**T**	**A**	**A**	N	**P**	**A**	**T**	**S**	**T**	**V**	**L**	**I**	**V**	**R**	D	**L**	**G**	**Y**	**A**	**Y**	
23	3	NGS	7.2	1.0E+05	7.3E-04	3.6	**G**	**R**	**T**	**F**	**S**	**S**	**Y**	**A**	**I**	**S**	**S**	**G**	**G**	**G**	N	**T**	**A**	**A**	N	**P**	**A**	**T**	**S**	**T**	**V**	**L**	**I**	**V**	**R**	D	**L**	**G**	**Y**	**A**	**Y**	
24	3	AI/ML	7.3	9.3E+04	6.9E-04	3.0	**G**	**F**	**T**	**F**	**S**	**S**	**Y**	**A**	**I**	**S**	**S**	**S**	**G**	**G**	**S**	**T**	**A**	**A**	N	**P**	**A**	**T**	**S**	**E**	**V**	**L**	**I**	**V**	**R**	D	**L**	**G**	**Y**	**A**	**Y**	
25	3	NGS	7.6	9.6E+04	7.3E-04	2.8	**G**	**F**	**T**	**F**	**S**	D	**Y**	**A**	**I**	**S**	**S**	**S**	**G**	**G**	**S**	**T**	**A**	**A**	N	**P**	**A**	**T**	**S**	**T**	**V**	**L**	**I**	**V**	**R**	D	**L**	**G**	**Y**	**A**	**Y**	
26	3	NGS	7.9	7.0E+04	5.5E-04	5.5	**G**	**F**	**T**	**F**	**G**	N	**Y**	**A**	**I**	**S**	**R**	**S**	**G**	**S**	**S**	**T**	**A**	**A**	N	**P**	**A**	**T**	**S**	**R**	**V**	**I**	**I**	**V**	**R**	D	**L**	**G**	**Y**	**A**	**Y**	
27	3	NGS	8.0	8.2E+04	6.6E-04	7.2	**G**	**L**	**T**	**F**	**S**	**S**	**Y**	**A**	**I**	**S**	**G**	**S**	**G**	**D**	N	**T**	**A**	**A**	N	**P**	**A**	**T**	**S**	**R**	**V**	**I**	**I**	**V**	**R**	E	**L**	**G**	**Y**	**A**	**Y**	
28	3	AI/ML	15.5	5.9E+04	9.1E-04	18.7	**G**	**F**	**T**	**L**	**S**	**D**	**Y**	**V**	**I**	**S**	**S**	**S**	**G**	**G**	N	**T**	**A**	**A**	N	E	**A**	**T**	**S**	**E**	**V**	**L**	**I**	**V**	**R**	D	**L**	**G**	**Y**	**A**	**Y**	
29	4	NGS	0.8	9.6E+04	8.0E-05	5.8	**G**	**R**	**T**	**L**	**G**	N	**Y**	**A**	**I**	**S**	**W**	**G**	**G**	**S**	**R**	**T**	**A**	**T**	**S**	**L**	**T**	**Y**	D	**Q**	**T**	**T**	**V**	**Y**	**V**	**S**	**P**	**L**	**A**	**Y**	**V**	D
30	4	NGS	1.3	1.5E+05	2.0E-04	5.0	**G**	**R**	**T**	**F**	**S**	N	**Y**	**A**	**I**	**S**	**G**	**G**	**G**	**G**	N	**T**	**A**	**T**	**S**	**L**	**T**	**Y**	D	**Q**	**T**	**T**	**V**	**Y**	**V**	**S**	**P**	**L**	**A**	**Y**	**G**	D
31	4	AI/ML	2.0	5.4E+05	1.1E-03	3.5	**G**	**R**	**T**	**L**	**S**	N	**Y**	**A**	**I**	**S**	**S**	**S**	**G**	**G**	**S**	**T**	**A**	**T**	**S**	**L**	**T**	**Y**	D	**Q**	**T**	**T**	**V**	**Y**	**V**	**S**	**P**	**L**	**A**	**Y**	**V**	D
32	4	AI/ML	2.1	4.1E+05	8.6E-04	4.6	**G**	**R**	**T**	**L**	**S**	N	**Y**	**A**	**I**	**S**	**S**	**G**	**G**	**S**	N	**T**	**A**	**T**	**S**	**L**	**T**	**Y**	D	**Q**	**T**	**T**	**V**	**Y**	**V**	**S**	**P**	**L**	**A**	**Y**	**V**	D
33	4	AI/ML	2.6	3.6E+05	9.2E-04	3.6	**G**	**R**	**T**	**F**	**S**	**S**	**Y**	**A**	**I**	**S**	**W**	**S**	**G**	**G**	**S**	**T**	**A**	**T**	**S**	**L**	**T**	**Y**	D	**Q**	**T**	**T**	**V**	**Y**	**V**	**S**	**P**	**L**	**A**	**Y**	N	N
34	4	NGS	2.8	4.5E+05	1.3E-03	3.8	**G**	**F**	**T**	**L**	**S**	N	**Y**	**A**	**I**	**S**	**S**	**S**	**G**	**D**	**S**	**T**	**A**	**T**	**S**	**L**	**T**	**Y**	D	**Q**	**T**	**T**	**V**	**Y**	**V**	**S**	**P**	**L**	**A**	**Y**	**V**	D
35	4	NGS	3.1	2.9E+05	9.2E-04	3.9	**G**	**F**	**T**	**F**	**S**	**D**	**Y**	**A**	**I**	**S**	**S**	**S**	**G**	**G**	N	**T**	**A**	**T**	**S**	**L**	**T**	**Y**	D	**Q**	**T**	**T**	**V**	**Y**	**V**	**S**	**P**	**L**	**A**	**Y**	**V**	D
36	4	NGS	3.4	4.0E+05	1.4E-03	3.3	**G**	**R**	**T**	**F**	**S**	**S**	**Y**	**A**	**I**	**S**	**S**	**S**	**G**	**G**	**S**	**T**	**A**	**T**	**S**	**L**	**T**	**Y**	D	**Q**	**T**	**T**	**V**	**Y**	**V**	**S**	**P**	**L**	**A**	**Y**	**V**	D
37	4	NGS	4.7	6.0E+04	2.8E-04	4.3	**G**	**F**	**T**	**F**	**S**	**S**	**Y**	**A**	**I**	**S**	**W**	**S**	**G**	**G**	**R**	**T**	**A**	**T**	**S**	**L**	**T**	**Y**	D	**Q**	**T**	**T**	**V**	**Y**	**V**	**S**	**P**	**L**	**A**	**Y**	N	N

**FIGURE 3:**
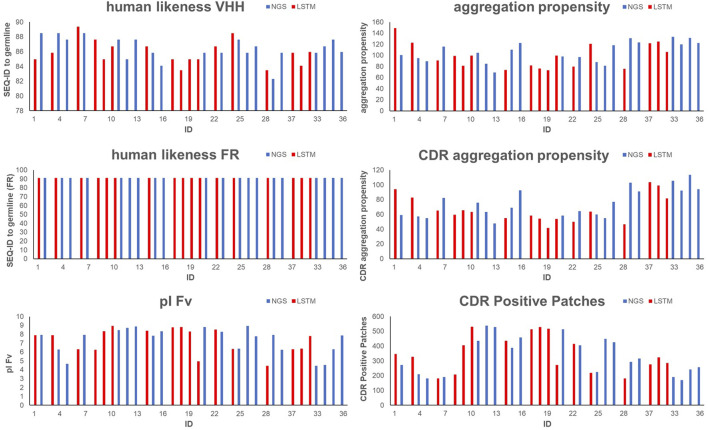
Graphical visualization of *in silico* properties for VHH domains that were selected for synthesis and experimental profiling. Blue bars indicate sequences obtained from NGS, red bars indicate sequences obtained from AI/ML (LSTM) sampling.

Table 3 and Supplementary Table S5 display the CDR1-3 sequences that were ultimately selected, along with their computed developability properties. For the specific rationale behind selecting each sequence for synthesis and experimental profiling, please refer to Supplementary Tables S1–S4. As shown in Supplementary Table S5 and Figure 3, due to our humanized VHH library design strategy all selected sequences show a high human-likeness in the framework region of 91.3%. Furthermore, due to our selection strategy, no sequence shows pronounced computed aggregation propensity or positive charged patches in the CDRs. However, as intended by the selection criteria, the sequences cover a certain diversity in NLL scores, pI, sequence diversity and chemical liability motifs.

### NGS and AI/ML derived sequences display high-affinity antigen binding and favorable early developability properties

**FIGURE 4:**
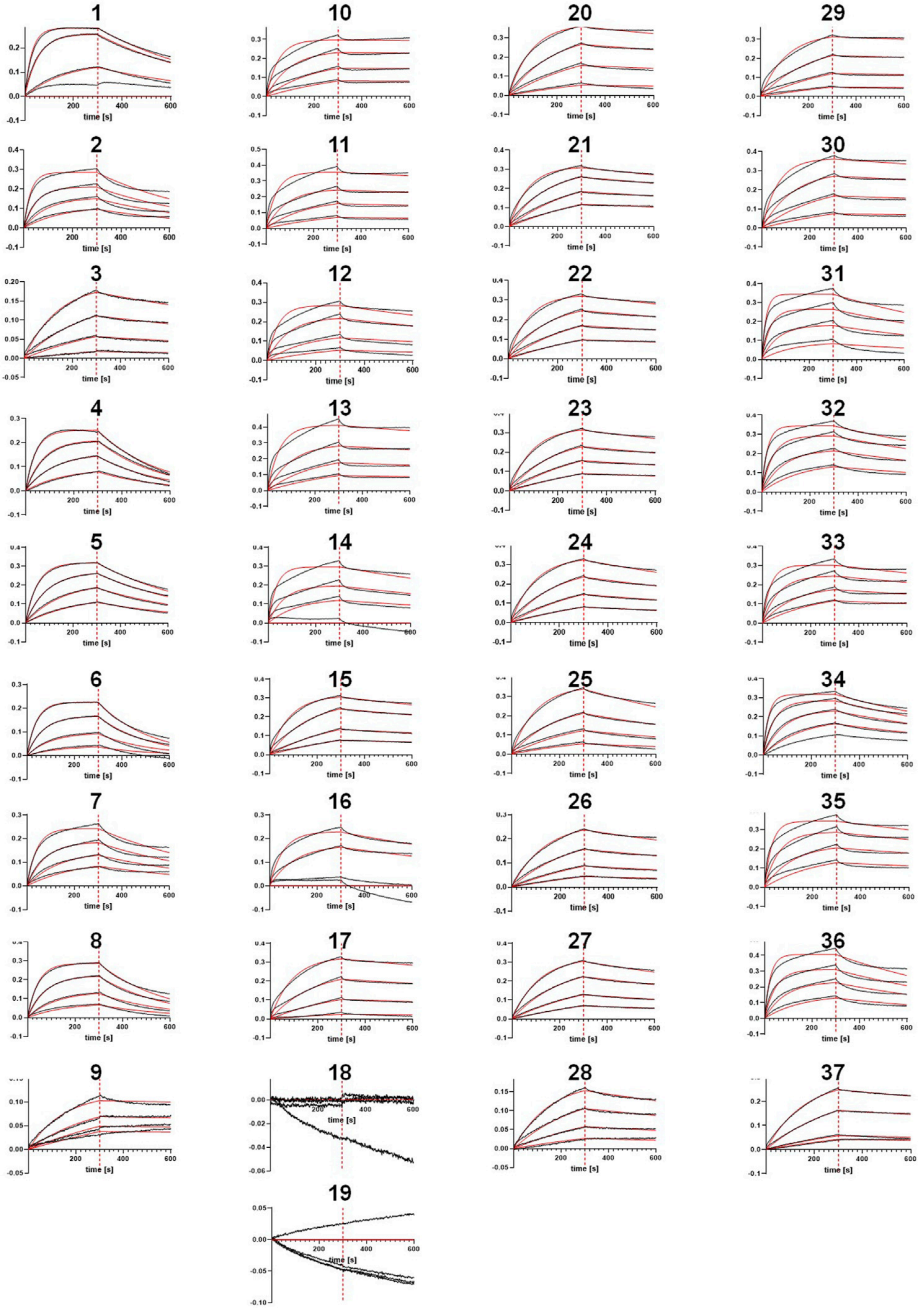
Bio-Layer Interferometry (BLI) curves (in black) and fitting curves (in red) obtained for all sequences.

As illustrated in Figure 1D, the selected sequences (Table 3) were utilized to synthesize one-armed, monovalent paratope-Fc fusion constructs as described previously (Klausz et al., 2022; Lipinski et al., 2023) to exclude avidity-related interactions that might enhance apparent binding affinity (Vauquelin and Charlton, 2013). For this, we utilized the strand-exchanged engineered domain (SEED) technology for Fc heterodimerization (Davis et al., 2010). Production was performed in ExpiCHO™ cells at a scale of 5 mL for experimental profiling. Expression yields were in the double-digit milligram-per-liter scale for most sequences, indicating adequate productivities for transient expression (Table 4). Furthermore, aggregation propensities as determined by analytical size-exclusion chromatography (SEC) post protein A purification indicated favorable biophysical properties for most sequences (Table 4). Binding experiments utilizing bio-layer interferometry (BLI) at varying (rh) NKp46 concentrations revealed specific antigen binding of the vast majority of tested VHHs from both approaches, NGS and AI/ML, respectively (Table 3; Figure 4). Encouragingly, within each sequence cluster, we obtained multiple sequences binding in the 1-digit nanomolar or even sub-nanomolar range to (rh) NKp46 (Table 3). Notably, although the affinity improvements are not significant, for three of the four sequence clusters, the most potent binder was obtained from the LSTM-predicted sequences, suggesting that the deep generative model approach can propose improved sequences in terms of binding affinities within the sequence space spanned by the NGS data set. Analysis of the NLL scores do not show a linear correlation to the experimentally observed binding affinities. However, within this specific dataset, high predicted (*i.e.*, unfavorable) NLL scores qualitatively translated to low or no detectable affinities, suggesting the use of more stringent NLL cutoff scores in future studies to eliminate true negatives from the list of candidates to be synthesized.

**TABLE 4:** Analytical and early developability data for selected one-armed VHH SEEDbodies and antibody controls, including amount of protein, SEC Purity, mean T_onset_, HIC retention time, AC-SINS and PSR-BLI.

ID	source	amount of protein [mg/L]	SEC Purity [%]	Mean Tonset [°C]	HIC tR [min]	AC-SINS [Δλmax (nm)]	PSR/BLI
1	AI/ML	49.1	92.7	59.1	4.9	-0.705	-0.011
2	NGS	51.8	90.9	59.2	5.0	-0.076	0.036
3	AI/ML	33.7	89.4	58.4	4.9	-1.189	0.004
4	NGS	28.0	96.9	58.1	4.9		
5	NGS	23.8	94.5	59.8	5.1	-0.550	0.016
6	AI/ML	25.2	96.5	58.1	5.1		
7	NGS	29.4	91.4	58.4	4.9	-0.570	0.030
8	AI/ML	32.2	95.0	59.8	5.3	-0.596	0.037
9	AI/ML	43.5	83.1	59.4	5.0	-0.550	0.005
10	AI/ML	26.5	97.1	59.1	4.8	-0.604	-0.031
11	NGS	23.7	97.3	58.9	4.8	-0.516	-0.012
12	NGS	18.1	100.0	58.0	4.8		
13	NGS	22.3	97.1	58.7	4.9	-0.578	0.021
14	AI/ML	25.1	99.2	57.4	4.9		
15	NGS	25.1	100.0	59.0	6.4	3.296	-0.011
16	NGS	19.5	98.8	57.4	5.4		
17	AI/ML	20.9	100.0	58.8	5.3	-0.497	0.007
18	AI/ML	50.9	98.8	58.7	5.2	-0.343	0.015
19	AI/ML	46.0	96.2	58.6	4.7	-0.548	0.015
20	AI/ML	36.8	99.1	56.3	5.0		
21	NGS	25.1	99.2	56.4	5.0		
22	AI/ML	37.7	98.3	58.2	5.2	-0.434	0.016
23	NGS	47.4	98.7	58.6	5.2	-0.504	0.035
24	AI/ML	34.9	97.3	58.5	5.1	-0.617	0.017
25	NGS	43.2	97.9	58.3	5.1	-0.511	0.024
26	NGS	30.7	97.4	58.0	5.0	-0.119	0.014
27	NGS	37.7	96.4	58.6	4.9	-0.310	0.039
28	AI/ML	22.3	82.2	58.5	5.0	-0.526	0.049
29	NGS	24.8	99.0	58.9	4.8	-0.395	0.019
30	NGS	37.2	100.0	58.4	6.0	-0.671	-0.020
31	AI/ML	133.2	99.1	58.7	6.4	-0.534	-0.011
32	AI/ML	71.6	100.0	59.0	6.4	-0.605	0.000
33	AI/ML	22.2	100.0	57.8	6.4		
34	NGS	33.1	100.0	58.1	6.3	-0.307	0.018
35	NGS	26.2	94.4	57.4	6.3		
36	NGS	48.2	97.8	58.6	6.7	-0.631	-0.009
37	NGS	22.2	97.1	58.7	6.5	-0.476	0.030
Trastuzumab						-0.001	0.009
Briakinumab				63.6		25.961	0.115
Avelumab					7.2		
Cetuximab					5.8		

**FIGURE 5:**
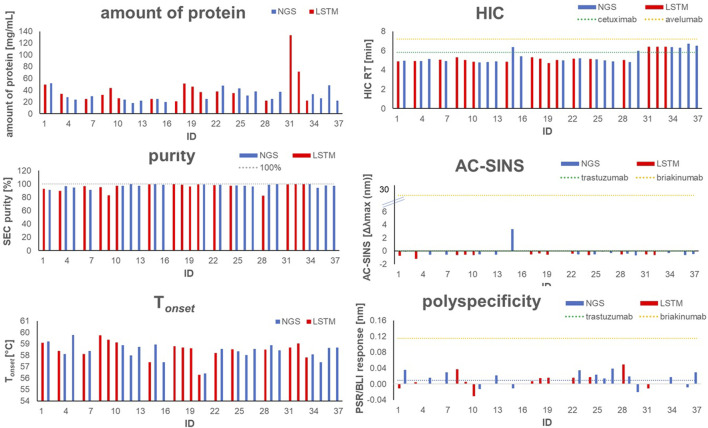
Graphical visualization of experimental analytical and early developability data for selected one-armed VHH SEEDbodies and antibody controls, including amount of protein, SEC Purity, mean T_onset_, HIC retention time, AC-SINS and polyspecificity (PSR-BLI). Blue bars indicate sequences obtained from NGS, red bars indicate sequences obtained from AI/ML (LSTM) sampling.

**FIGURE 6:**
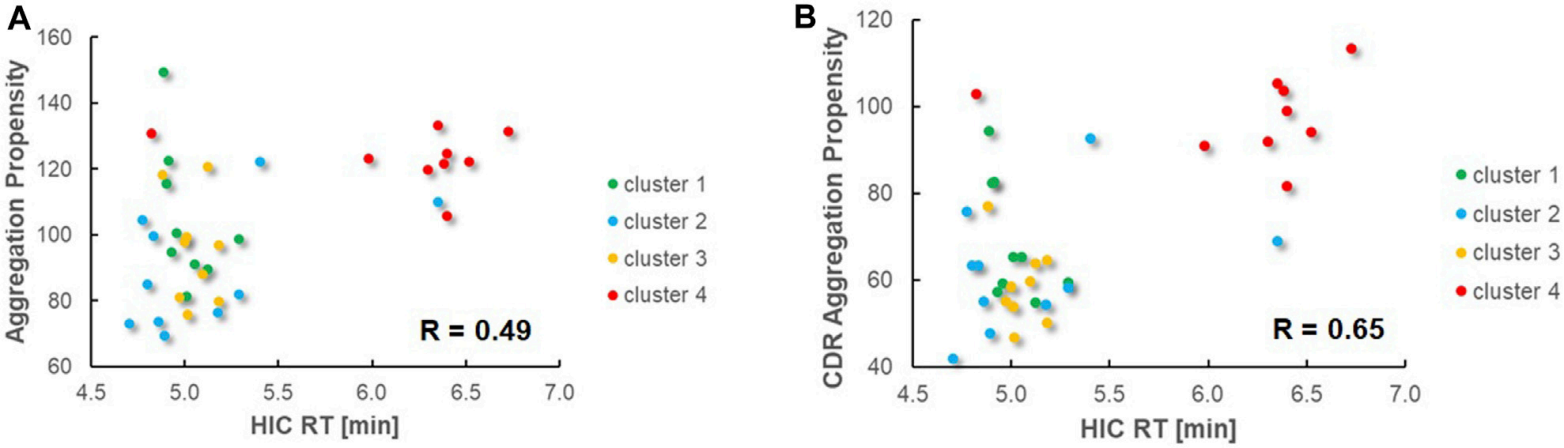
Comparison of predicted aggregation propensities vs. experimental HIC retention times and Pearson correlation values. Sequences from different clusters are shown in different colors. **(A)**. Predicted aggregation propensities based on the entire variable VHH regions. **(B)**. Predicted aggregation propensities based on the CDR regions only.

To experimentally assess early developability properties (Table 4; Figure 5), we exploited analytical size-exclusion chromatography (SEC) after protein A purification as a first filter. Generally, purities above 85% target peak are considered as adequate attributes for transient antibody expression, while purities of more than 90% indicate favorable properties. Overall, most sequences showed a high target purity above 90%. As additional early developability attribute we also scrutinized one-armed VHH SEEDbodies using analytical hydrophobic interaction chromatography (HIC) assuming that a low overall hydrophobicity would contribute to a good developability profile. For this, we utilized two marketed therapeutic antibodies as assay controls, cetuximab and avelumab, with HIC retention times of 5.8 min and 7.2 min, respectively. Overall, HIC retention times of the vast majority of VHH SEEDbodies were in the lower favorable range. In this respect most molecules displayed even shorter retention times compared to cetuximab, indicating a beneficial (low) relative hydrophobicity of the VHH domains. Only variants of CDR3 cluster 4 (IDs 30–37) showed retention times in the range of 6.0–6.7 min that are in between the ones from cetuximab and avelumab. Notably, although there is no ideal linear correlation between HIC retention times (Table 4) and computed aggregation propensities (Supplementary Table S5 and Figure 6), these *in silico* scores are (in agreement with their higher retention times) on average higher for IDs 30–37 (cluster 4) compared to the other sequences; supporting their usefulness for early *in silico* ranking and filtering of sequences. The observed degree of correlation between predicted and experimental hydrophobicity is in agreement with a recent systematic study on antibody structures (Waibl et al., 2022). Based on that study, prediction accuracy for HIC retention scales might be further improved by i) exploring alternative approaches for 3D model generation and by i) using hydrophobicity scales derived from experimental HIC data.

To further investigate the biophysical properties of the herein identified VHHs, we checked the thermostability of the molecules by nanoDSF. The T_*onset*_ of a dedicated molecule represents the temperature where the variable domain of a VHH construct starts to unfold while applying a temperature gradient and as such, is an indicator of its thermostability in a certain buffer and pH environment. The T_*onsets*_ we measured were in the range between 56°C and 59 °C for all tested molecules, representing an overall adequate thermostability for further development (Mieczkowski et al., 2023). As obvious from Figure 5, no significant differences in T_onset_ are observed between the sequences obtained from NGS and LSTM sampling, supporting the claim that LSTM is capable of correctly modeling long-term dependencies and capturing relationships between amino acids that determine structure and function. Additionally, we evaluated available VHH SEEDbodies (that were selected based on remaining substance availability) in affinity-capture self-interaction nanoparticle spectroscopy (AC-SINS) as early experimental predictor for colloidal stability (Liu et al., 2014). Clinical antibody trastuzumab was used as assay control indicating favorable biophysical properties with mean Δλmax values of ∼0.2 nm after subtraction of buffer blanks. Final AC-SINS scores for the tested VHH SEEDbodies were calculated via subtraction of blank and trastuzumab scores (Table 4). The calculated scores indicate favorable colloidal stability properties for all tested SEEDbodies, very similar to trastuzumab and significantly better compared to briakinumab, which was used as reference with a known propensity for self-interaction (Jain et al., 2017). As further early developability assessment, the selected SEEDbodies were evaluated in the polyspecificity reagent (PSR) assay which provides insights into the general off-target interactions/specificity and selectivity of the VHH domains, again using trastuzumab as indicator for reduced unspecific interactions and briakinumab reference indicating more pronounced polyspecificity (Table 4). Compared to these assay controls, no SEEDbody shows pronounced non-specific binding.

Although we have to keep in mind that the monospecific IgG1 control antibodies might not be ideal references for benchmarking our one-armed VHH SEEDbodies, the available experimental data indicate favorable intrinsic developability properties for the VHH domains.

To experimentally assess the risk for the formation of chemical degradation products along the drug development process, which might potentially affect its efficacy and safety, one potent sequence from each of the CDR3 clusters was subjected to forced oxidation and deamidation studies (Nowak et al., 2017) (Table 5; see Materials and Methods for experimental details and Supplementary Table S6 for detailed experimental results). Within the CDR regions of the four selected sequences, we could only observe significant deamidation within CDR1 of sequence **1**, attributed to the Asn-Tyr sequence motif. This non-canonical motif is generally known as non-highly susceptible to deamidation (Lu et al., 2019), but in the present case this chemical liability is a potential critical quality attribute (CQA) that would require additional efforts for monitoring and control in the development process. The SAR data shown in Table 3 demonstrate that several alternative sequence variants with similar potency are available, which are devoid of this chemical liability motif and might be selected as alternative optimized hits. This example illustrates the benefit that the explicit selection of sequence variants within specific CDR3 clusters provide valuable SAR data that do not only point to mutations that finetune binding affinity, but also to optimize the physico-chemical property profiles (regarding chemical liabilities, PTMs, electrostatic and hydrophobic properties).

**TABLE 5:** Deamidation and oxidation modifications observed within CDR1-3 in accelerated oxidation and deamidation studies, shown as % modified species after 24 h vs. 0 h. CDR residues that are typically prone to Asn-deamidation N) or Met-oxidation (M) are colored in red. ND: no degradation detected.

ID	CDR1								CDR2								CDR3																				CDR1% modified	CDR2% modified	CDR3% modified
1	G	R	T	F	S	N	Y	A	I	S	R	G	G	D	N	T	A	A	V	F	T	P	T	D	T	V	V	F	I	N	K	E	P	Y	N	Y	18.7	<1	ND
10	G	G	T	F	G	S	Y	A	I	S	R	S	G	G	S	T	A	A	A	G	G	M	G	S	T	T	V	V	V	S	T	I	P	Y	K	Y			ND
22	G	R	T	F	S	S	Y	A	I	S	S	S	G	G	N	T	A	A	N	P	A	T	S	T	V	L	I	V	R	D	L	G	Y	A	Y			<1	<1
30	G	R	T	F	S	N	Y	A	I	S	G	G	G	G	N	T	A	T	S	L	T	Y	D	Q	T	T	V	Y	V	S	P	L	A	Y	G	D	<1	<1	

### AI/ML derived sequences fill gaps within the sequence space spanned by NGS data

**FIGURE 7:**
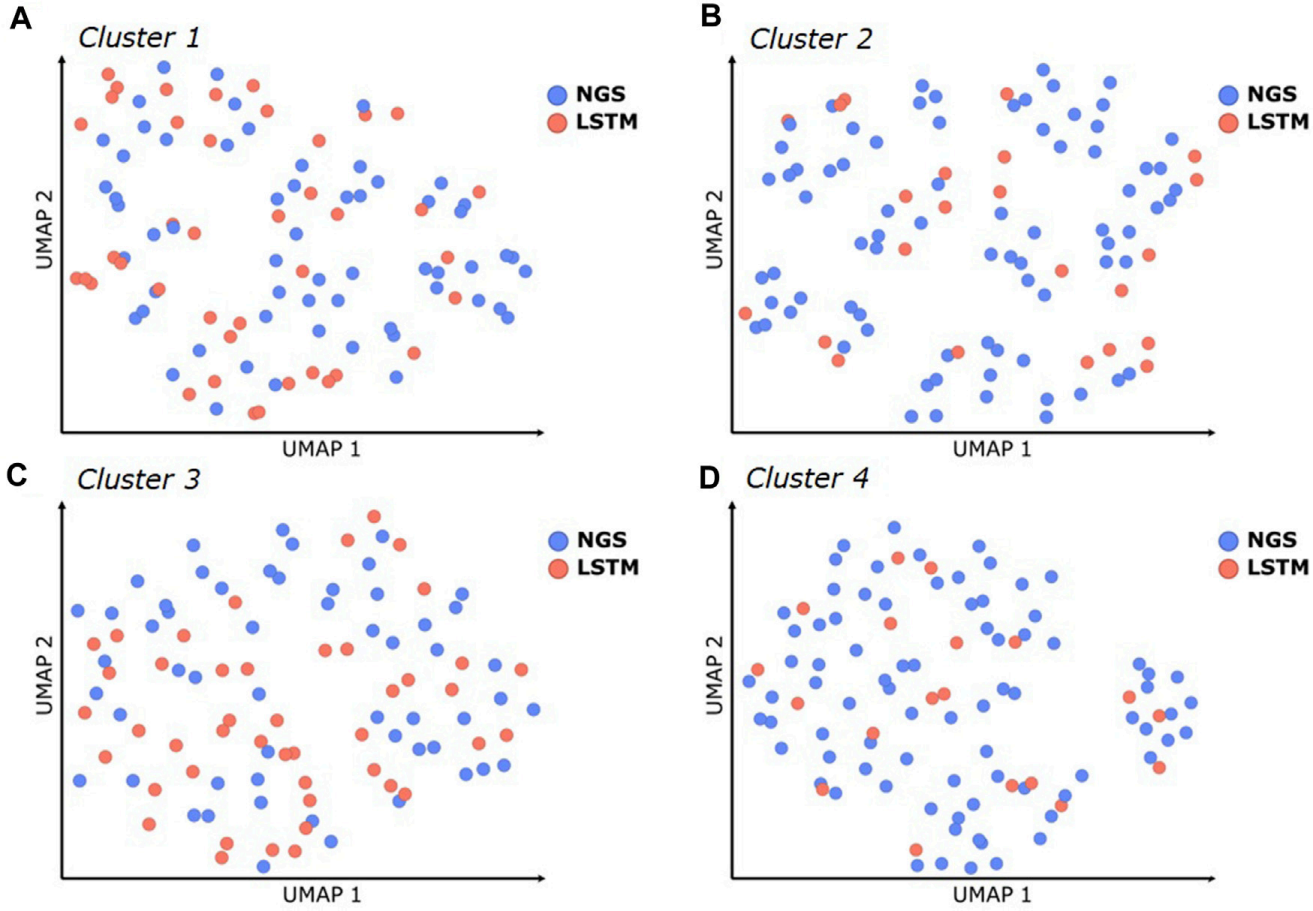
Similarity of CDR1-3 sequences within the best 100 scoring sequences (based on their NLL) for each CDR3 sequence cluster **(A–D)**, illustrated using UMAP dimensionality reduction. Blue dots represent sequences that were obtained from NGS, red dots represent new sequence combinations that were automatically designed with LSTM.

The experimental results demonstrate that several optimized hit sequences were obtained within each cluster, suitable for further project progression, including experimental characterization in functional assays, early formulation studies, and/or *in vivo* experiments. These sequences were derived from both the NGS data and the LSTM sampled sequences. To investigate the benefit of LSTM sampling, we analyzed the number and diversity of additional unique sequences designed by LSTM in comparison to the NGS sequences. Our analysis focused on the top-ranked 100 NLL scorers within each CDR3 cluster, since all tested variants from these lists showed favorable binding affinities (Supplementary Tables S1–S4). As illustrated in a UMAP dimension reduction based on sequence diversity, the LSTM approach generated a considerable number of new sequence combinations, effectively filling gaps within the sequence space spanned by the NGS dataset (see Figure 7 and the underlying sequences in Supplementary Tables S1–S4), thereby increasing not only the number of potent sequences but also the likelihood of including variants with lower risks of chemical degradation or post-translational modification motifs. For CDR3 cluster 1, 41 of the top 100 sequences were obtained from LSTM (cluster 2: 23, cluster 3: 45, cluster 4: 19). The predicted physical properties (pI, hydrophobicity/aggregation propensity, CDR Positive Patches) of the LSTM sampled sequences covered a similar range and diversity as those obtained from NGS (see Supplementary Figure S8). Moreover, a comparative inspection of production yield, melting temperatures, and other biophysical properties (Figure 5) between the LSTM and NGS-derived sequences that had been synthesized did not reveal any significant differences. This finding supports the claim that LSTM sampling can enrich the pool of NGS sequences with additional potent and developable binders, which increases the overall chance of discovering optimized hits with favorable developability profiles.

## Discussion

In the past, the discovery and optimization of antibodies and VHHs were predominantly reactive in nature (Evers et al., 2023b): Traditional screening methods were used to obtain antibody or VHH sequences, which were subsequently sequence-optimized with regards to factors such as binding affinity, human-like characteristics, and chemical stability. Following the identification of the top-performing optimized hits, developability assessments were carried out. These assessments, since conducted after sequence optimization, aimed to identify any suboptimal developability characteristics, such as aggregation, low solubility, poor expression, non-specific binding, or unfavorable pharmacokinetic properties. Consequently, issues arising from these suboptimal properties were passed on to downstream functions, e.g., Drug Metabolism and Pharmacokinetics (DMPK), non-clinical safety, and Chemistry, Manufacturing and Controls (CMC) to adjust and optimize downstream process development and dosing regimens, thereby often imposing delays in development, increased costs and finally a considerable risk for the project to achieve approval for First in Human and further clinical studies (Evers et al., 2023b). To mitigate these risks, in this work we propose an integrated and efficient *de novo* design strategy comprising camelid immunization, library generation, YSD, FACS, NGS analysis, AI/ML methods, *in silico* developability assessment as well as synthesis and early experimental characterization of the selected sequences. In an ideal scenario, these subsequent steps can be accomplished in less than 4 months without the need for subsequent time-consuming steps of iterative sequence optimization. This comprehensive approach was successfully applied for an early drug discovery project to generate automatically humanized and sequence optimized VHH binders against NKp46 with favorable early developability profiles.

The *in silico* steps described in this study are computationally inexpensive (<1 week in this study) and can be combined into a fully automated workflow. Furthermore, our process of CDR3 engraftment upon camelid immunization onto a generic humanized and sequence-optimized scaffold library is characterized by its low complexity and duration (<1 week). Besides camelid VHH library generation, we have established a similar CDR grafting approach for the generation of ultralong CDR-H3 antibodies following the immunization of cattle (Pekar et al., 2021). Since finally NGS is meanwhile quick and cost-effective, the herein described combination of experimental and *in silico* approaches represent a general strategy for a fast and efficient hit discovery and optimization upon camelid immunization. An alternative option that bypasses animal immunization and thereby can even further accelerate the *de novo* identification of developable antibodies or VHHs is the screen of diverse synthetic libraries that were tailored towards human-likeness and favorable physico-chemical properties (Teixeira et al., 2021; Khetan et al., 2022; Evers et al., 2023b). Binders obtained from antibody selections and NGS analysis of such diverse libraries might further be optimized towards improved binding and developability applying AI/ML approaches as described in the present study. As recently discussed (Gray et al., 2020; Gray et al., 2020; Custers and Steyaert, 2020; Laustsen et al., 2021), both animal immunization and synthetic library technologies have their own benefits and drawbacks for antibody discovery. For example, while synthetic libraries bypass the need of animal immunization, the immune system of animals has evolved over millions of years to efficiently produce highly specific antibodies against a diverse range of antigens. The semi-immune/semi-synthetic procedure presented in this study combines the advantages of both technologies and is coupled with the benefits of NGS and AI/ML approaches for rapid and efficient antibody discovery and optimization (Laustsen et al., 2021).

In this study, we opted for a LSTM, a recurrent neural network (RNN) architecture, as the basis of sequence prediction models based on NGS data. The selection of this approach was based on the fact that it has been successfully applied to diverse modalities (Saka et al., 2021; Müller et al., 2018; Gupta et al., 2018; Merk et al., 2018; Segler et al., 2018; Z et al., 2022) and that the code was already available (Müller et al., 2018). From a scientific perspective, LSTM models are known for their capability to learn complex patterns and dependencies within sequences. Therefore, by training on existing protein sequences from NGS data, the LSTM can capture essential structural and functional motifs present in the library, potentially generating new functional sequence combinations not observed in the NGS dataset. The experimental data from the present study confirmed that the LSTM sampled sequences did not exhibit significant disadvantages compared to the NGS-derived sequences in terms of production yield, melting temperatures, or binding affinities. Various other ML approaches have also demonstrated effectiveness for the identification of complex patterns from sequence input data and were successfully employed for antibody design based on NGS data (Liu et al., 2020; Mason et al., 2021; Makowski et al., 2022; Hu et al., 2023; Li et al., 2023; Parkinson et al., 2023). Furthermore, additional deep generative modelling methods such as variational auto-encoders (VAEs) and generative adversarial networks (GANs) may also be explored to optimize sequence spaces obtained from NGS data (Akbar et al., 2022).

LSTM sampling efficiently filled diversity gaps in the sequence space beyond what is covered by the NGS training data (Figure 7). However, since the present LSTM approach uses one-hot amino acid encoding, it will generate new sequence combinations that only interpolate within the sequence space covered by the NGS data. Therefore, another aspect that might be further investigated is the representation of amino acids in the context of *in silico* sequence processing. Most approaches utilize one-hot encoding, which does not capture structural features, inherent relationships, or the physicochemical similarities between amino acids. Several alternative encoding schemes, such as amino acid embeddings, physicochemical descriptors or position-specific scoring matrices (PSSMs) might be suited to increase the model’s ability to extrapolate into new sequence spaces.

Another crucial aspect for AI/ML based prediction and identification of improved binders is the scoring function used to rank the sequences based on their assumed binding affinity against the target. In this study, we utilized NLL that assumes a correlation of binding affinity with the observed amino acid distribution in the NGS set of sequences obtained after FACS. Notably, the majority of synthesized VHH constructs (>80%) exhibited binding affinities in the (sub-)1-digit nanomolar range. Therefore, based on the limited experimental data from this study, we consider the NLL ranking as the suited criterion for selecting sequences with a high likelihood of binding. For a more comprehensive conclusion, future systematic studies would be required to explore correlations with other scoring functions for identifying high-affinity binders. However, such analyses would necessitate a large dataset of sequences with experimental binding affinity data.

Recent studies have already shown the successful application of AI/ML techniques on antibody NGS data to design new sequences with even further improved potency or developability (Liu et al., 2020; Mason et al., 2021; Saka et al., 2021; Makowski et al., 2022; Hie et al., 2023; Parkinson et al., 2023). While these studies focused on optimizing previously identified antibody candidates through sequence diversification and library generation, the present study represents, to the best of our knowledge, the first prospective application of AI/ML for the *de novo* identification of diverse, potent, and developable VHHs. In contrast to these previous studies, our approach was applied on a humanized library that originated from a highly diverse camelid repertoire upon immunization.

To validate the efficacy of our approach, we conducted experimental profiling to assess binding affinity and developability for multiple sequences per cluster and gained valuable SAR and SPR information directly from the initial set of synthesized variants. This procedure mirrors the well-established “hit-triaging” approach for small molecules obtained from high-throughput screens, where multiple molecules within different chemical series are evaluated to identify the most promising candidates for further development (Kitchen and Decornez, 2015). As an advantage, this procedure can directly point to lead molecules and backups without the need for additional time-consuming sequence optimization cycles.

The present study represents a first successful application of our integrated VHH discovery approach on NKp46 as specific target. Further ongoing and future studies on internal projects will demonstrate the robustness of this process and certainly point to aspects that may be further optimized, e.g., regarding the design of a follow up humanized VHH scaffold library (Arras et al., 2023), *in silico* property predictors and further aspects as described above.

Finally, the findings and results of this study should also be considered in the light of some limitations and inspirations for further future studies (Jin et al., 2023). In the present study, we applied a CDR3 sequence identity cutoff of 50% for sequence clustering as a compromise to find i) sequences within one cluster that all bind in a similar mode to the same epitope and ii) at the same time provide sufficient sequence diversity for SAR analysis and automated multi-parameter sequence optimization. It is generally known that similar protein sequences have similar folds (Baker and Sali, 2001). However, if this is also true for CDR3 loops and whether the 50% cutoff is the most ideal cutoff for this purpose will require additional dedicated studies (Könning et al., 2017). One might question the general need for LSTM sampling if the sequences obtained from NGS analysis of the semi-immune/semi-synthetic strategy are already “good” enough. The present study demonstrates that i) high affinity binders with favorable early developability profiles can already be obtained from data mining of the available NGS data, but in addition ii) that LSTM sampling is able to fill sequence gaps with additional potent and developable sequences that have not obtained from the NGS data. The timeframe for LSTM model generation and sequence sampling (<1 day in the present study) is negligible in the context of a standard hit discovery campaign. Therefore, our general recommendation is to add the LSTM-based designs alongside NGS-derived sequences. Then, select the best binders from the combined pool based on their predicted likelihood of binding and relevant *in silico* developability parameters, aligned with the specific target product profile (TPP) of the project. This approach enhances the overall project success probability (Krah et al., 2016). To ensure proper assay controls for early experimental developability assessments, we used four well-characterized monospecific IgG1s (avelumab, cetuximab, trastuzumab, and briakinumab) as references. While these control sequences allow assay comparisons across different studies, they may not serve as ideal benchmarks for drawing final conclusions about the general developability of our VHHs, since we fused them to SEED Fc domains that show considerable sequence differences to IgG1 Fc domains. As a conclusion, the data presented in this study only indicate favorable intrinsic developability properties for the VHHs generated here. Further in-depth studies, including the identification and use of specific VHH-based controls for benchmarking, will be necessary to assess how these developability properties extend to different multi-specific architectures (Bannas et al., 2017; Chanier and Chames, 2019; Pekar et al., 2020; Yanakieva et al., 2022; Lipinski et al., 2023; Wang et al., 2022) Quality of NGS data is critical for any AI prediction tool, as it forms the basis for training. In this study, we used NGS data obtained from different round of FACS. As we learned through the course of the study, sample preparation, read depth, sequence complexity and sequencing error rates can significantly impact the results. The rate of enrichment over FACS round 2 vs. round 0 was used as an essential parameter for nominating sequence clusters, but this enrichment was biased due to the low number of reads in round 0, and the final selection might have varied based on variations in NGS data generation and analysis. Nevertheless, the reads used for LSTM sampling after FACS round 2 were sufficiently broad and frequent to discover potent binders with favorable early developability profiles.

In conclusion, the herein presented workflow comprising a combination of AI/ML methods, camelid immunization, library generation, NGS analysis, and *in silico* developability assessment can identify potent VHH binders with promising early developability profiles. This singular procedure mitigates the need for subsequent sequence optimization, thereby offering the potential to significantly accelerate hit discovery and optimization and at the same time to reduce the risk for developability-related attrition in the downstream process.

## Materials and Methods

### NGS, sequence clustering and ranking

To prepare RNA material for NGS analysis, two defined antisense primer sequences were used which specifically aligned with nucleotides in the upper hinge regions of camelid IgG2 and IgG3 antibody isotypes, facilitating directed cDNA synthesis. Within a subsequent PCR utilizing index primers for Illumina sequencing, the VHH sequences were amplified and tagged. For the samples derived from the VHH diversities embedded in the plasmid vector system, the sequences processed accordingly, but lacking the cDNA synthesis step. During the DNA amplification process, the AMPure system (Beckman Coulter) was used to purify the VHH amplicons, while for the purification of the final sequencing library a Pippin Prep (Sage Science) was used. For sequencing purposes, a MiSeq (Illumina) device with the v3 600 cycle kit according to the manufacturer’s protocol was employed. Resulting FASTQ files were uploaded to Geneious Biologics (https://www.geneious.com/biopharma) for analysis and annotation. Reads were overlapped, filtered for length, and the VHH sequences were annotated using the *Lama glama* reference library. Normalized counts for each CDR3 were used to identify sequences that were enriched in the sorted samples relative to the baseline diversity.

Sequences were clustered based on 50% CDR3 sequence identity. All sequence clusters were assessed and ranked by their i) NGS counts after the second FACS round and their ii) enrichment (“Fold Change”) over round 0 to 2. The enrichment factor EF (“Fold Change”) was calculated according to the following formula:

$$EF=\frac{{\left(\frac{N_{cluster}+1}{N_{total}+1}\right)}_{S2}}{{\left(\frac{N_{cluster}+1}{N_{total}+1}\right)}_{S0}}$$

Where N represents the number of reads within the specific cluster and S0, S2 represent the FACS selection round.

### LSTM model structure, training and sampling

The code from Müller et al. (Müller et al., 2018) (https://github.com/alexarnimueller/LSTM_peptides) has been used and slightly adapted to constrain the input training sequence length to the length of CDR1-CDR2-CDR3 output sequences of the individual clusters. The adapted code and the sequences used as input for training and sampling of new sequences are available from https://github.com/MCompChem/LSTM_CDRs. The input sequences had been exported from Geneious Biologics as csv file and used as input sequences without further preprocessing. Sequences are represented in one-hot encoding scheme, in which a one-hot residue represents a single amino acid (single letter code). The LSTM architecture was chosen based on hyperparameters described by Saka et al. (2021). The chosen network architecture for this study was a two-layer LSTM recurrent neural network consisting of 64 neurons and a 0.2 dropout rate and trained for 200 epochs. Remaining parameters were set to default values as described by Müller et al. were utilized for all other parameters in the network. Based on five-fold cross validation, the epoch with the best average performance were chosen for the given LSTM architecture for each cluster individually. For each cluster, 10,000 sequences were sampled from the selected best epoch model.

### Likelihood for sequence ranking

The NLL (negative log-likelihood) is a statistical measure that describes the likelihood of observing each amino acid at each position within the set of sequences over a training data set. From a set of sequences, the NLL is computed for each sequence according to the following formula:

$$NLL=-\sum_{k=1}^{K}\ln p\left(x_{k}\right)$$

where $p\left(x_{k}\right)$ represents the generative probability of observing a residue *x* at the k -th position of the sequence and K is the sequence length.

### *In silico* developability assessment

The *in silico* developability profiles were computed using an internal pipeline termed “Sequence Assessment Using Multiple Optimization Parameters (SUMO)” (22). This approach automatically generates VHH models based on the provided sequences of the variable regions, identifies the human-likeness by sequence comparison to the most similar human germline sequence, determines structure-based surface-exposed chemical liability motifs (unpaired cysteines, methionines, asparagine deamidation motifs and aspartate deamidation sites) as well as sites susceptible to post-translational modification (N-linked glycosylation). Moreover, a small set of orthogonal computed physico-chemical descriptors including the isoelectric point (pI) of the variable domain, Schrodingers AggScore as predictor for hydrophobicity and aggregation tendency calculated for the complete variable domain as well as the complementarity-determining regions (CDRs) only and the calculated positive patch energy of the CDRs were determined (Sankar et al., 2018). These scores were complemented with a green to yellow to red color coding, indicating scores within one standard deviation from the mean over a benchmarking dataset of multiple biotherapeutics approved for human application as green, scores above one standard deviation as yellow and those above two standard deviations as red (Ahmed et al., 2021). For the AggScore values, these cutoffs were slightly adjusted based on correlation analyses to internal experimental HIC data.

### Protein expression and analysis

The sdAb variants were integrated into the pTT5 mammalian expression vector by fusing them at the hinge region of Fc immune effector-silenced (eff-) SEED AG chains (Thermo Fisher Scientific). This fusion allowed the generation of one-armed (oa) SEEDbodies, using a SEED-GA chain without paratope.

The proteins were produced using the ExpiCHO™ Expression System (Thermo Fisher Scientific) in either 5 or 25 mL scale, following the standard protocol provided by the manufacturer. The expression was carried out with a 2:1 ratio of AG to GA chain. After 7 days of expression, the supernatants containing the proteins were purified using MabSelect™ antibody purification chromatography resin (Cytiva) using 20 mM acetic acid followed by an neutralization (500 mM sodium phosphate buffer, 1.5 M NaCl, pH 8) to a final formulation pH of 6.8 in PBS. The purified proteins were then subjected to sterile filtration, and their concentrations were determined by measuring the absorbance at 280 nm (A_280_).

To evaluate the monomer content of the protein samples, analytical size-exclusion chromatography (SEC) was performed. Each sample contained 7.5 µg of protein and was run on a TSKgel UP-SW3000 column (2 μm, 4.6 × 300 mm, Tosoh Bioscience) using an Agilent HPLC 1260 Infinity system. The mobile phase consisted of 50 mM sodium phosphate and 0.4 M NaClO4 at pH 6.3, with a flow rate of 0.35 mL/min. The signals were recorded at 214 nm.

For assessing the hydrophobicity of the different molecules, hydrophobic interaction chromatography (HIC) was employed. Each sample contained 20 µg of protein and was analyzed on a TSKgel Butyl-NPR column (2.5 µm, 4.6 × 100 mm, Tosoh Bioscience) using an Agilent HPLC 1260 Infinity system with a flow rate of 0.5 mL/min. Prior to injection, the samples were mixed with a 50% (v/v) solution of 2 M ammonium sulfate. A gradient was applied, running from mobile phase A (1.2 M ammonium sulfate in PBS) to mobile phase B (50% methanol in 0.1x PBS) over a period of 15 min at 25°C. Signals were recorded at 214 nm. The reference molecules, anti-PD-L1 Avelumab and anti-EGFR Cetuximab, were used for comparison.

To investigate the thermal unfolding properties of the antibodies, differential scanning fluorimetry (DSF) was performed using a Prometheus NT. PLEX nanoDSF instrument (NanoTemper). The samples were measured in duplicate using nanoDSF Standard Capillary Chips. A temperature gradient ranging from 20°C to 95°C at a slope of 1°C/min was applied. Fluorescence signals at 350 nm and 330 nm were recorded. The unfolding transition midpoints (Tm) and Tonset values were determined from the melting curves or the first derivative of the fluorescence ratio 350 nm/330 nm.

### Bio-Layer Interferometry (BLI)

The biophysical properties of the sdAbs were evaluated using an Octet Red BLI system from Sartorius. The binding experiments were conducted in KB-buffer (PBS pH 7.4, 0.1% BSA, 0.02% Tween-20) using Protein G Biosensors. The biosensors were loaded with the one-armed antibody samples at a concentration of 3 μg/mL for 180 s. The samples were subjected to a 2-fold serial dilution of (rh) NKp46 (ACRO Biosystems), starting at a concentration of 100 nM using a measurement window of 300 s for association and dissociation each.

The obtained data was aligned to the association step, and inter-step correction was applied during the dissociation step. To reduce noise, Savitzky-Golay filtering was employed. The resulting data were analyzed using a 1:1 binding model to determine the binding kinetics and affinity between the binders and (rh) NKp46.

### Forced oxidation and deamidation studies

Forced protein oxidation was introduced to the samples (30 μg, 1 mg/mL) by diluting with an equal volume of 0.1% H_2_O_2_ (Merck, 107,209) and incubation at room temperature. After 0, 6, and 24 h a 10 µL aliquot was taken and the oxidation reaction was stopped by buffer exchange to 25 mM NH_4_HCO_3_ (Merck, 101131), pH 7 with Amicon filter devices (Merck, UFC503096), respectively. To force protein deamidation 30 µg sample was buffer exchanged to 25 mM NH_4_HCO_3_ (Merck, 101131), pH 10 using Amicon filter devices (Merck, UFC503096). Subsequently, the sample volume was adjusted to 30 µL and incubated at 37°C. To stop the deamidation reaction the sample was buffer exchanged to NH_4_HCO_3_, pH 7 as described previously in the oxidation workflow.

#### Peptide mapping

Proteins were unfolded and reduced by addition of 5 µL 12 M Urea (Merck, 108487) and 1 µL 50 mM DTT (Merck, 111474) and subsequent incubation at 50 °C for 30 min. Reduced samples were then alkylated by addition of 2.5 µL 55 mM iodoacetamide (Merck, 804744) and incubation at room temperature for 30 min in the dark. Samples were then mixed with 30 µL 25 mM NH_4_HCO_3_ and 3 µL trypsin solution (0.1 mg/mL). After 6 h at 37°C, 0.5 µL 50% FA was added and the peptides were analyzed by LC-MS. LC-MS analysis was performed using an Exion HPLC system (Buffer A: 0.1% formic acid in water (Biosolve, 23244101), Buffer B: 0.1% formic acid in acetonitrile (Biosolve, 01934101)) coupled to a Sciex 6,600+ mass spectrometer by a Turbo V ESI source. 8 μg peptide solution was loaded onto an Aeris 1.7 µm PEPTIDE XB-C18 150 × 2.1 mm column (Phenomenex, 00B-4506-AN) and eluted with a linear gradient from 5% to 50% Buffer B within 49 min and 0.25 mL/min flow rate. Data were acquired in IDA mode with positive polarity, in a mass range from 230 to 1,600 m/z. Other instrument settings were as follows: source voltage 5.5 kV, declustering potential 80 V, accumulation time 0.25 s, source temperature 450°C, maximum number of candidate ions per cycle 10, gas1 45 L/h, and gas2 45 L/h. The mass spectrometer was calibrated with ESI positive calibration solution 5,600. Acquired data were processed with Genedata Expressionist 16.5. Chemical noise subtraction was applied to the data by clipping all data points below an intensity of 50. Furthermore, spectra were smoothed, and background subtracted. For peptide mapping the MS tolerance was 20 ppm and the MS/MS tolerance 0.1 Da. Trypsin was chosen as enzyme with maximum 2 missed cleavages and minimum 3 amino acid peptide length. Deamidation (NQ), glutamine to pyroglutamate conversion, c-terminal lysine loss, and oxidation (MW) were selected as variable modifications.

#### AC SINS

Molecules were captured onto particles via immobilized capture antibodies and self-association was judged in PBS buffer at pH 7.4 by shifts in the plasmon wavelengths (Makowski et al., 2021). Clinical antibody Trastuzumab was used as control indicating favorable biophysical properties with mean Δλmax values of ∼0.2 nm after subtraction of buffer blanks. Final AC-SINS scores for molecules were calculated via subtraction of blank and Trastuzumab scores and the calculated scores of the molecules in the range of −0.46 and 0.06 indicate favorable developability properties very similar to Trastuzumab.

#### PSR-BLI

To assess non-specific antibody interactions to polyspecificity reagent (PSR), a published cytometric assay (Xu et al., 2013) was adapted for the application of fast and sensitive Bio-Layer Interferometry (BLI). PSR was derived from soluble membrane proteins (SMP) of CHO and HEK293-6E cells as described by Xu et al. (2013). Assays were performed at 25°C with orbital sensor agitation at 1,000 rpm in 200 µL volume with DPBS. Pre-hydrated AHC biosensors were loaded with antibody (10 μg/mL) for 300 s. Afterwards biosensors were blocked with 1% BSA for 200 s and a baseline was established by rinsing in DPBS for 60 s. Association with 20 μg/mL PSR (1:1 mixture of CHO and HEK293-6E SMP) was performed for 100 s. As reference, association was performed in DPBS. To calculate the PSR-BLI score, the binding response from the association step was normalized to the reference measurement by subtraction, followed by subsequent subtraction with non-loading control (DPBS).

## Data Availability

The original contributions presented in the study are included in the article/Supplementary Materials. The adopted python code and the sequences that had been used for LSTM model generation are available from https://github.com/MCompChem/LSTM_CDRs. Further inquiries can be directed to the corresponding author.
