# Supplementary material for: AI/ML combined with next-generation sequencing of VHH immune repertoires enables the rapid identification of de novo humanized and sequence-optimized single domain antibodies: a prospective case study
Source: Front Mol Biosci. 2023 Sep 28;10:1249247. doi: 10.3389/fmolb.2023.1249247 (PMC10575757; doi:10.3389/fmolb.2023.1249247)
Supplement: Supplementary file 7 [file DataSheet1.docx]

**Supplementary Information: AI/ML combined with Next Generation Sequencing of VHH immune repertoires enables the rapid identification of *de novo* humanized and sequence-optimized single domain antibodies: a prospective case study.**

Paul Arras^1,2^, Han Byul Yoo^1^, Lukas Pekar^1^, Thomas Clarke^3^, Lukas Friedrich^4^, Christian Schröter^5^, Jennifer Schanz^5^, Jason Tonillo^5^, Vanessa Siegmund^6^, Achim Doerner^1^, Simon Krah^1^, Enrico Guarnera^1^, Stefan Zielonka^1,2^and Andreas Evers^1*^

^1^Antibody Discovery & Protein Engineering, Merck Healthcare KGaA, Frankfurter Straße 250, D-64293 Darmstadt, Germany

^2^Institute for Organic Chemistry and Biochemistry, Technical University of Darmstadt, Alarich-Weiss Straße 4, D-64287 Darmstadt

^3^Bioinformatics, EMD Serono, 45 Turnpike, Billerica, MA 01821, USA

^4^Computational Chemistry and Biologics, Merck Healthcare KGaA, Frankfurter Straße 250, D-64293 Darmstadt, Germany

^5^ADCs & Targeted NBE Therapeutics, Merck KGaA, Frankfurter Str. 250, D-64293 Darmstadt, Germany

^6^Early Protein Supply & Characterization, Merck Healthcare KGaA, Frankfurter Straße 250, D-64293 Darmstadt, Germany

^*^To whom correspondence should be addressed:

Andreas Evers, E-mail: [Andreas.evers@merckgroup.com](mailto:Andreas.evers@merckgroup.com)


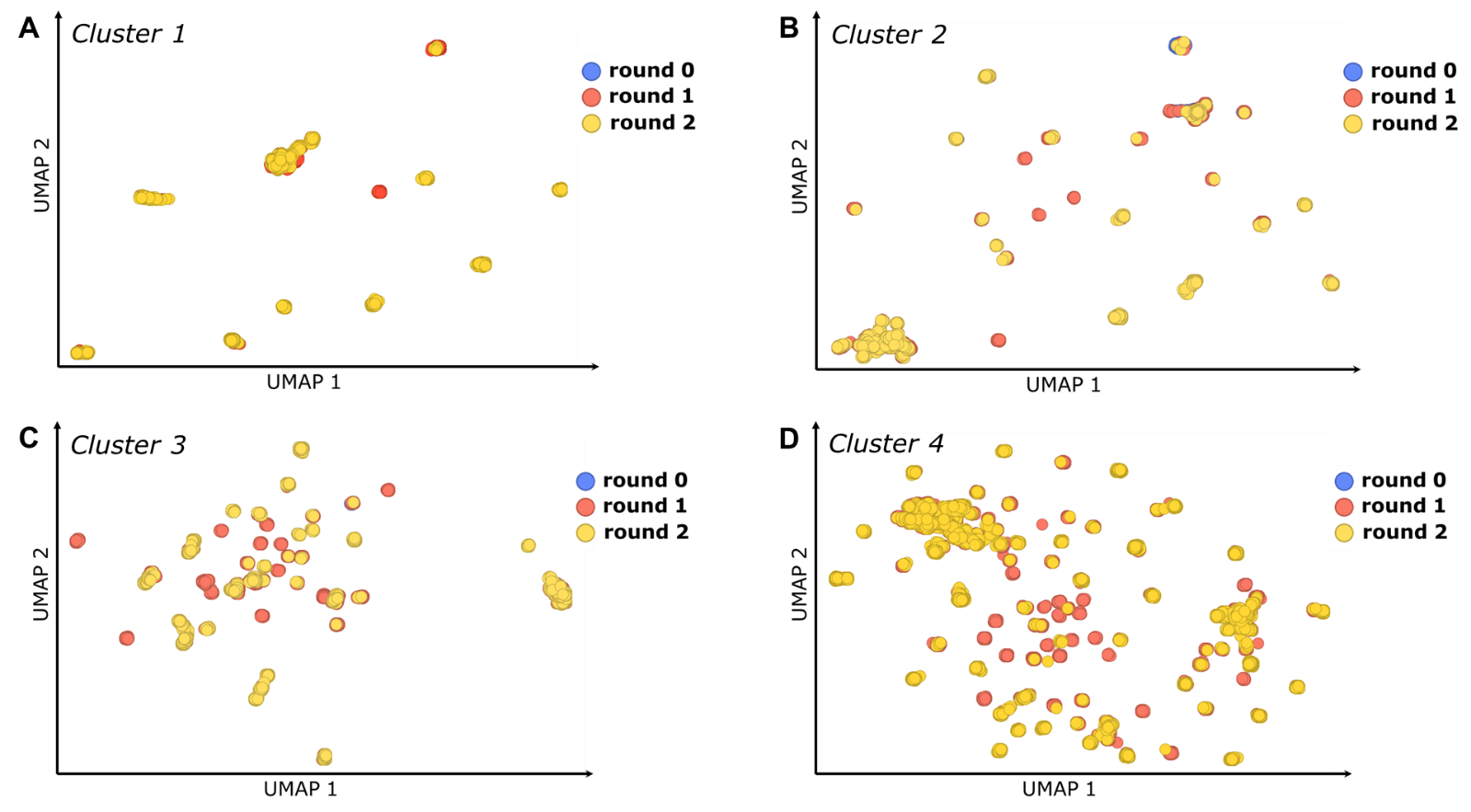


**Supplementary Figure 1**. Similarity of CDR1-3 sequences within the selected four CDR3 sequence clusters (A-D), obtained from different rounds of FACS (Table 1), illustrated using UMAP dimensionality reduction. Each dot represents the CDR1-3 of individual VHH sequences. Dots are colored based on round of FACS. Sequences from FACS round 0 are shown in blue, from round 1 in red and those from round 2 in yellow. Note that a considerable fraction of blue and red dots are hidden behind the yellow dots and therefore not visible.

**
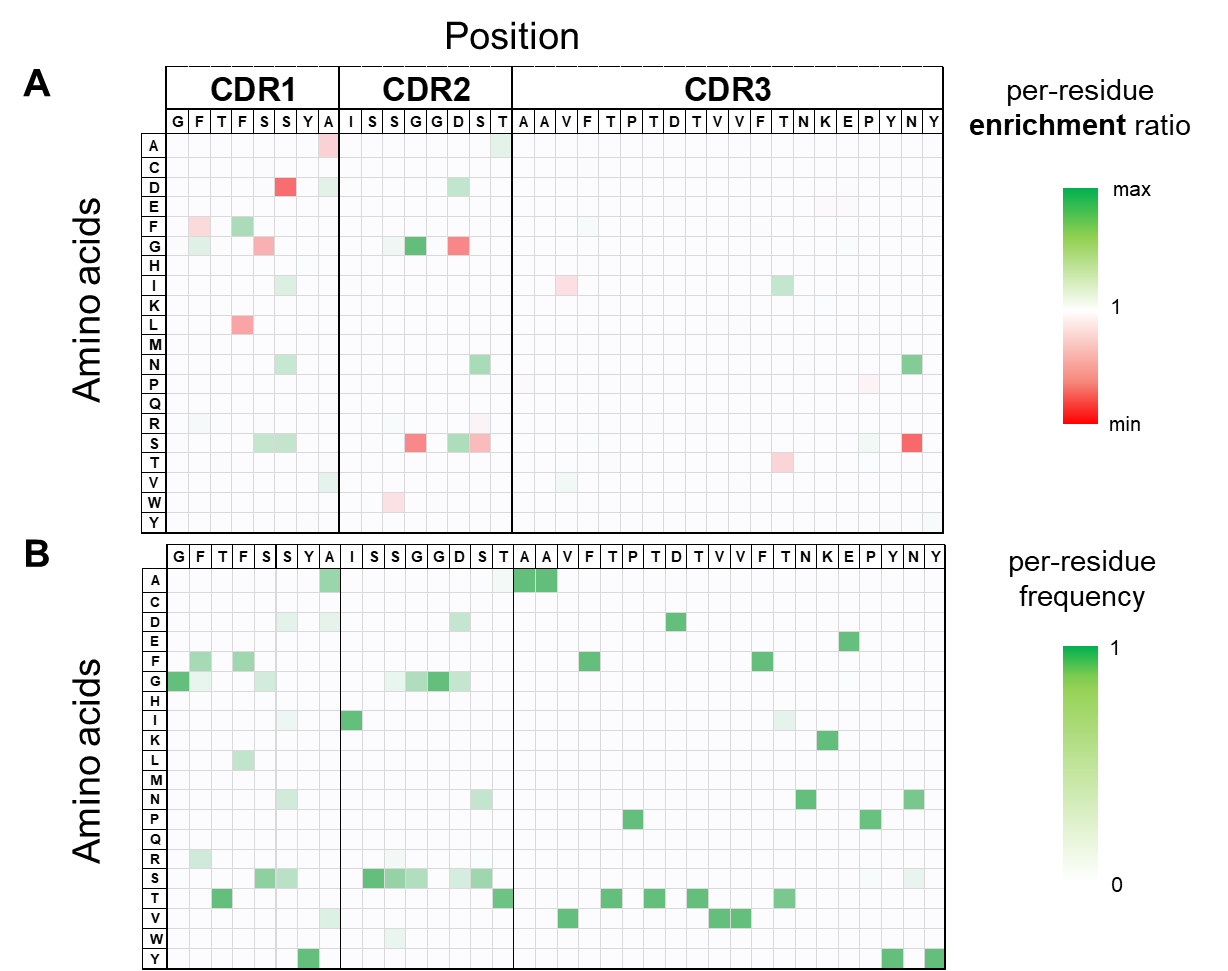
**

**Supplemental Figure 2**. Per-residue enrichment and frequency analysis, both illustrated as heat-map for CDR3 sequence cluster 1. The table headers show the CDR1-3 sequence of the most frequent clone observed in the NGS data set after the second round of FACS selection within this cluster. A. Per-residue enrichment ratio over YSD-FACS rounds 1-2. Residues with a high enrichment (colored green) are observed with a higher relative frequency after FACS round 2 compared to round 1. B. Per-residue frequency distribution observed after FACS round 2.


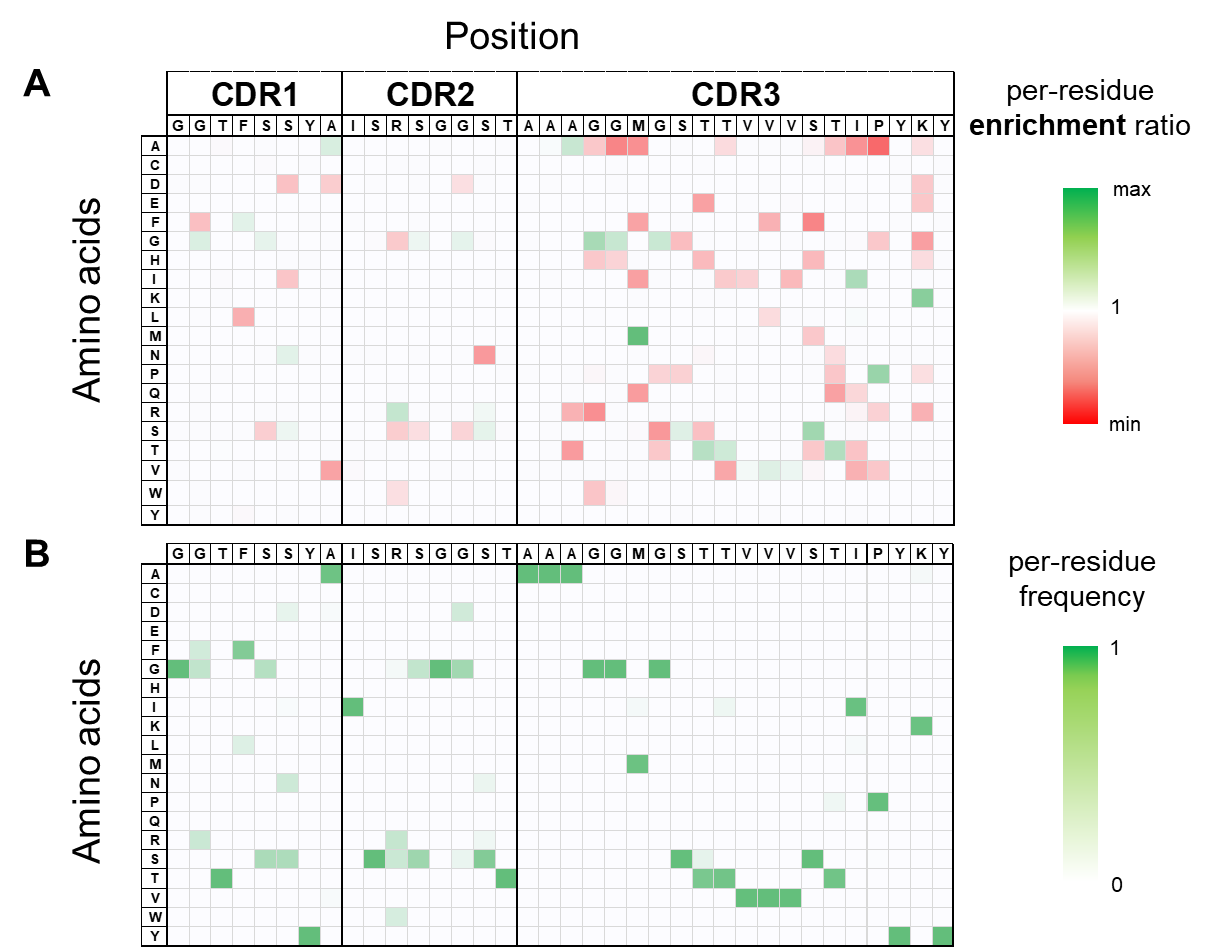


**Supplemental Figure 3**. Per-residue enrichment and frequency analysis, both illustrated as heat-map for CDR3 sequence cluster 2. The table headers show the CDR1-3 sequence of the most frequent clone observed in the NGS data set after the second round of FACS selection within this cluster. A. Per-residue enrichment ratio over YSD-FACS rounds 1-2. Residues with a high enrichment (colored green) are observed with a higher relative frequency after FACS round 2 compared to round 1. B. Per-residue frequency distribution observed after FACS round 2.


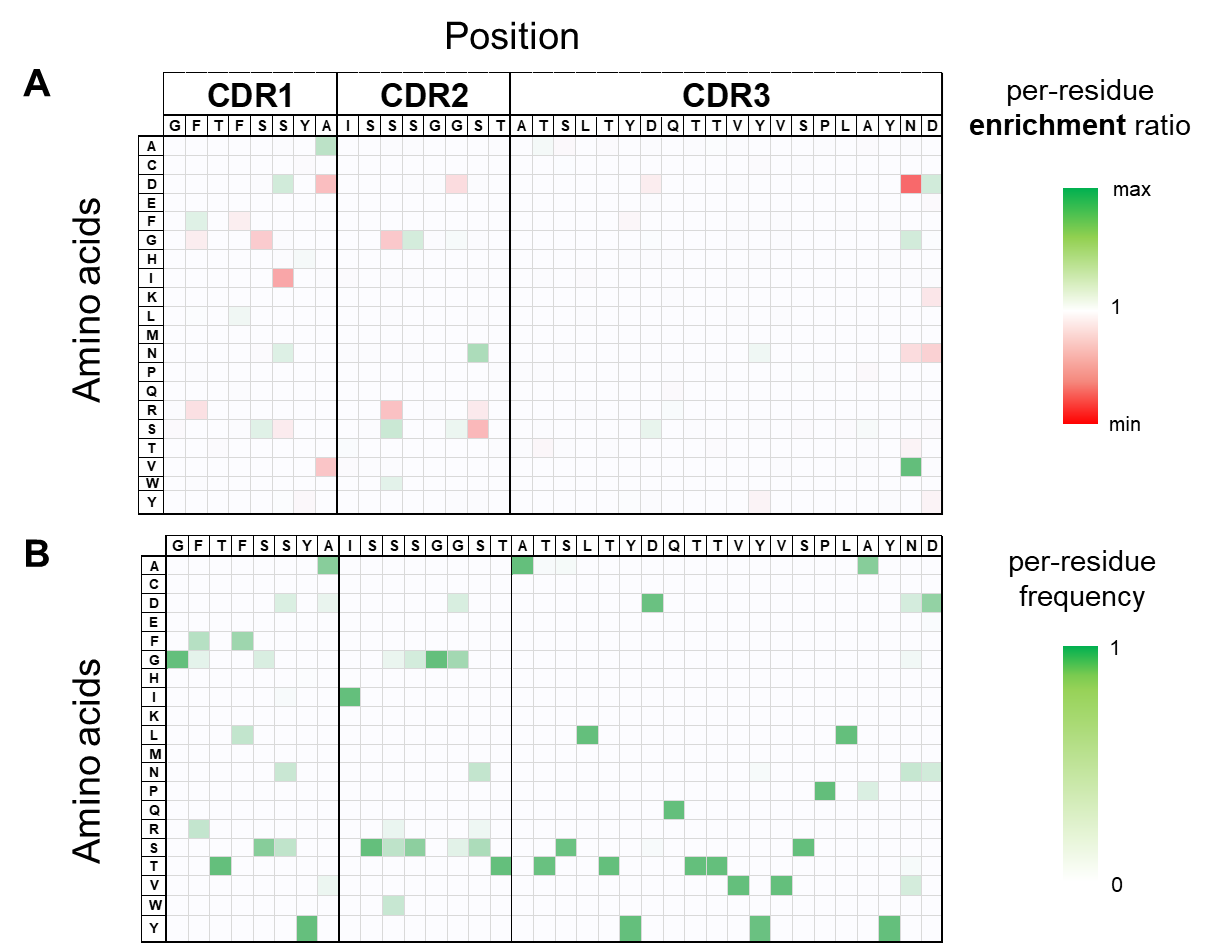


**Supplemental Figure 4**. Per-residue enrichment and frequency analysis, both illustrated as heat-map for CDR3 sequence cluster 4. The table headers show the CDR1-3 sequence of the most frequent clone observed in the NGS data set after the second round of FACS selection within this cluster. A. Per-residue enrichment ratio over YSD-FACS rounds 1-2. Residues with a high enrichment (colored green) are observed with a higher relative frequency after FACS round 2 compared to round 1. B. Per-residue frequency distribution observed after FACS round 2.

**Supplemental Figure 5**. Visualization of top-ranked NLL scorers from the combined pool of sequences obtained from NGS analysis of sequence pools from (i) the second round of FACS (labeled “S2” in the first column) and (ii) LSTM sampled sequences (labeled “sample”). In the sequence view of the CDR1-3 sequences, amino acid differences to the top-ranked NLL scorer (column label “NLL”) are shown in yellow. Visual inspection was done for the top-ranked 100 sequences to inspect in how far the LSTM-sampled sequences generate new sequence combinations that were not obtained from the NGS dataset. The data files containing this information for each CDR3 cluster are provided as separate Supplemental tables.

**Supplemental Figure 6**. Visualization of in silico developability profiles of top-ranked NLL scorers from the combined pool of sequences obtained from NGS analysis of sequence pools from (i) the second round of FACS (labeled “S2” in the second column) and (ii) LSTM sampled sequences (labeled “sample”). Analysis of this table was used in combination with the sequence view (Supplemental Figure 6) to select sequences based on SAR and SPR analysis, i.e. picking variants where new residue combinations resulted in specific variations of predicted phys-chem properties (mainly focusing on pI and potential chemical degradation sites). The data files containing this information for each CDR3 cluster are provided as separate Supplemental tables.


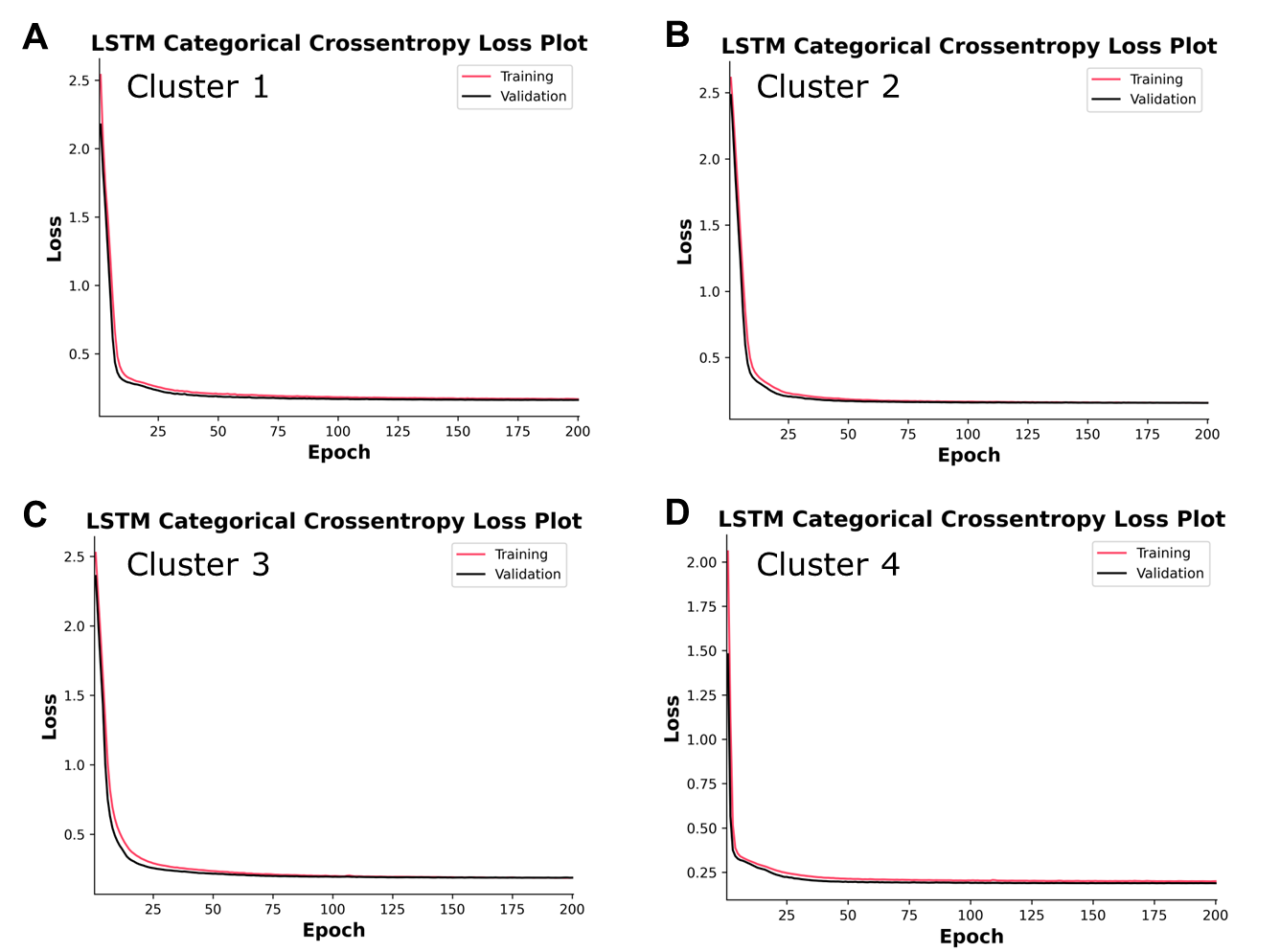


**Supplementary Figure 7**. Loss evaluation of training and validation datasets for the selected CDR3 clusters (A-D). Training; red, Validation; black.


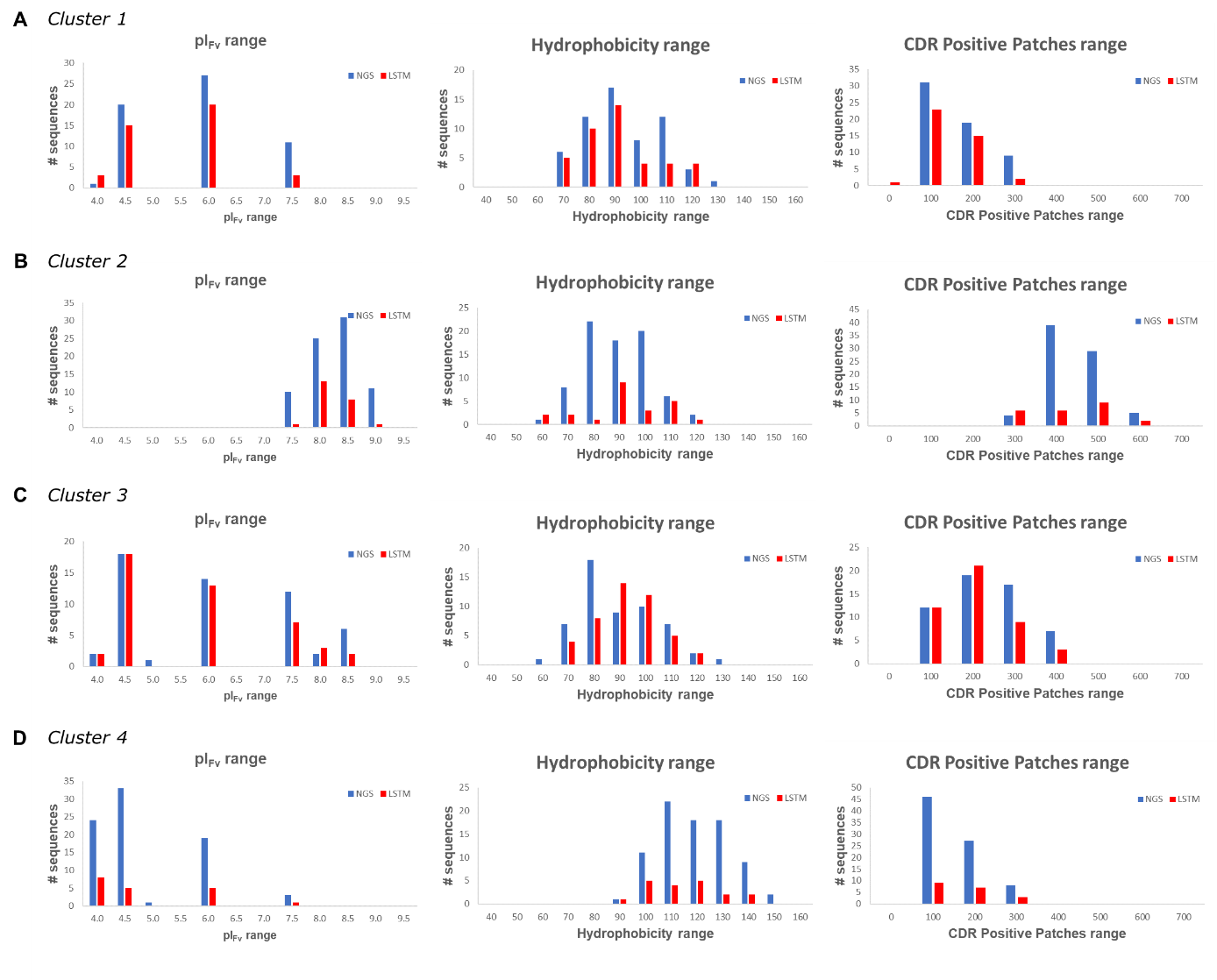


**Supplemental Figure 8**. Similarity of *in silico* developability properties (pI, hydrophobicity/aggregation and CDR positive patches) of NGS- and LSTM-derived best 100 scoring sequences (based on their NLL) for each CDR3 sequence cluster (A-D). Blue bars represent sequences that were obtained from the library approach (NGS), red bars represent new sequence combinations that were automatically designed with LSTM.

SupplementalTable1.xlsx

SupplementalTable2.xlsx

SupplementalTable3.xlsx

SupplementalTable4.xlsx

**Supplemental Tables 1-4.** These tables contain the following sequence and their computed property data for CDR3 cluster 1 (SupplementalTable1.xlsx), CDR3 cluster 2 (SupplementalTable2.xlsx), CDR3 cluster 3 (SupplementalTable3.xlsx) and CDR3 cluster 4 (SupplementalTable4.xlsx). The sheets entitled “ALL” list all sequences obtained after FACS round 2 (labelled “S2”) and the 10000 LSTM sampled sequences (labelled “sample”). In addition, the computed NLL scores are provided as well as a flag to indicate unique sequences (“unique”) and the information about the relative rank according to the NLL score. The sheets entitled “top100 SUMO” show the sequences of the different framework and CDR regions, the full variable region sequence and the computed *in silico* developability properties. The sheets entitled “final selection and rationale” show the CDR1-3 sequences of those molecules selected for synthesis and experimental profiling, together with a label about their origin (AI/ML or NGS), their measured K_D_ values, NLL scores, *in silico* developability properties and a brief explanation about the rationale for their selection (“selection rationale”). Finally, the sheets “top 100 properties” provide a comparative visual analysis (NGS- vs LSTM-derived sequences) of the best ranked 100 sequences (according to NLL) regarding their computed developability property distributions that are displayed in Supplemental Figure 8.

**Supplemental Table5.xlsx**

**SupplementalTable 5**. *In silico* developability assessment of VHH sequences obtained from NGS analysis and AI/ML (LSTM) predictions. VHHs were analyzed for their sequence identity compared to the most similar human germline (MOST SIMILAR GERMLINE) either based on the entire variable domain region (SEQ-ID) or the framework region only (SEQ-ID FR) as well as for their total number of specific chemical liabilities and PTMs, i.e., non-canonical cysteines, methionine oxidations, asparagine deamidations or aspartate isomerizations, and N-glycosylations, in structurally exposed CDR residues as derived from automatically generated models. As calculated physico-chemical developability descriptors (PHYS-CHEM), structure-based pI values (pI), computed aggregation propensities of the entire variable regions and the CDR regions only, as well as Positive Patches of the CDRs are shown. The complementing color coding indicates scores within one standard deviation from a benchmark mean (dataset of 77 antibodies approved for human application) as green, scores above one standard deviation as yellow and scores above two standard deviations as red. The mean (and standard deviation) values over this dataset are: (i) pI: 7.64 (1.2), (ii) Aggregation Propensity: 120.1 (48.9), CDR Aggregation Propensity: 67.5 (40.6), CDR Positive Patches: 421.3 (144.6). Of particular significance, the color coding employed in this study is established upon cutoff scores extracted from the variable regions of classical therapeutic heavy-light chain antibodies. It is important to note that, owing to the limited number of approved single-domain antibodies, definitive cutoff scores for VHHs are presently indeterminate. As a result, corresponding cutoff scores for the distinct VHH domains of caplacizumab and ozoralizumab are provided as *in silico* controls.

**Supplemental Table6.xlsx**

**SupplementalTable 6**. Detailed experimental results of forced deamidation and oxidation analysis of IDs 1, 10, 22, 30. The experimental details are described in Materials and Methods.
